# Air Pollutants’ Concentrations Are Associated with Increased Number of RSV Hospitalizations in Polish Children

**DOI:** 10.3390/jcm10153224

**Published:** 2021-07-22

**Authors:** August Wrotek, Artur Badyda, Piotr O. Czechowski, Tomasz Owczarek, Piotr Dąbrowiecki, Teresa Jackowska

**Affiliations:** 1 Department of Pediatrics, The Centre of Postgraduate Medical Education, 01-813 Warsaw, Poland; 2 Department of Pediatrics, Bielanski Hospital, 01-809 Warsaw, Poland; 3 Faculty of Building Services Hydro- and Environmental Engineering, Warsaw University of Technology, 00-653 Warsaw, Poland; 4 Polish Federation of Asthma Allergy and COPD Patients’ Associations, 01-604 Warsaw, Poland; pdabrowiecki@wim.mil.pl; 5 Department of Quantitative Methods and Environmental Management, Faculty of Management and Quality Science, Gdynia Maritime University, 81-225 Gdynia, Poland; oskar@am.gdynia.pl (P.O.C.); t.owczarek@wpit.umg.edu.pl (T.O.); 6 Department of Allergology and Infectious Diseases, Military Institute of Medicine, 04-141 Warsaw, Poland

**Keywords:** respiratory syncytial virus, air pollution, particulate matter, nitrogen dioxide, bronchiolitis, pneumonia, children, hospitalization

## Abstract

Respiratory syncytial virus (RSV) contributes significantly to pediatric hospitalizations. An association between air pollution and an increased number of RSV cases has been suggested. We sought to evaluate the short-term impact of air pollutants on RSV hospitalizations in Polish children in the period 2010–2019. Daily concentrations of PM_10_ and PM_2.5_ (particulate matter with an aerodynamic diameter less than or equal to 10 μm and 2.5 μm, respectively) and nitrogen dioxide (NO_2_) were analyzed in general regression models (GRM) to establish their influence and full interaction scheme. Significant seasonal and annual periodicity among 53,221 hospitalizations was observed; finally, data from the 2012–2019 RSV high-risk seasons created models for seven agglomerations. The addition of PM_2.5_, PM_10_, and NO_2_ to the basic model for RSV seasonality explained 23% (4.9–31%, univariate model) to 31.4% (8.4–31%, multivariate model) of the variance in RSV hospitalizations. A 10 μg/m^3^ increase in PM_2.5_, PM_10_, and NO_2_ concentrations was associated with 0.134 (0.087–0.16), 0.097 (0.031–0.087), and 0.212 (0.04–0.29) average increases in hospitalizations, respectively. In the multivariate models, PM_2.5_, PM_10_, and NO_2_ alone, as well as PM_2.5_–NO_2_, PM_2.5_–PM_10_, and PM_10_–NO_2_ interactions, were associated with hospitalizations in some of the locations, while the metaregression showed statistically significant interactions between each of the pollutants, and between the pollutants and the year of the study. The inclusion of PM_2.5_, PM_10_, and NO_2_ in GRM explains a significant number of RSV hospitalizations. The pollutants act alone and interact together in a varied manner. Reducing air contamination might decrease the costs of hospital healthcare.

## 1. Introduction

Respiratory syncytial virus (RSV) is one of the major etiological factors of upper and lower respiratory tract infections (LRTI). The latter often result in hospitalization, generating high costs for healthcare systems, mostly in the youngest group of patients [1,2,3]. The true frequency of RSV infections remains unknown, since up to one quarter of RSV infections may be asymptomatic in children under 2 years of age [4], but a number of symptomatic patients seek healthcare and/or require hospital treatment [4]. Global estimates report a causative role of RSV in approximately 20% of respiratory tract infections [5], and RSV-specific antibodies may be found in over 90% of children at the age of 3 [4]. Globally, RSV may be causing as many as 33 million LRTI episodes a year, and it leads to 3.2 million hospitalizations in children under 5 years of age [3].

RSV-related morbidity seems to be underestimated in other age groups than the pediatric age group; however, awareness on the impact of RSV in adults is increasing, and estimates report at least 10% of pneumonia cases in adults to be RSV-related [6,7]. Efforts towards RSV prevention have resulted in passive immunoprophylaxis with monoclonal antibodies administered to high-risk patients, while clinical trials on possible vaccines are still being conducted with no product licensed to date [8,9]. The risk factors of a more severe clinical course of the disease have mainly been studied in hospital settings, and include prematurity, younger age, and comorbidities related to pulmonary or cardiac conditions, but regarding the risk of hospitalization, air pollution is of special interest [10,11,12,13,14]. Low environmental air quality in community-based studies predisposes the population to the development of RSV LRTI, and there is increasing evidence of a negative effect of air pollutants on LRTI, hypothesizing that its harmful influence exceeds the influence of respiratory viruses [15]. In-depth analyses have focused on the attention to particulate matter characterized by an aerodynamic diameter no greater than 10 μm and 2.5 μm (PM_10_ and PM_2.5_), sulfur dioxide (SO_2_), nitrogen dioxide (NO_2_), and carbon monoxide (CO). According to the literature, the increased risk for RSV-related hospitalizations or emergency department consultations has been associated with exposure to PM_10_ [10,11,12,13,14], PM_2.5_ [13,16,17], benzene [18], NO_2_ [11,13], ozone [19], or SO_2_ [11], yet in the latter case, there are also contradictory reports [13]. Fewer analyses focused on RSV morbidity in an outpatient setting, but a large-scale Chinese study by Qing Ye confirmed a correlation with PM_2.5_, PM_10_, SO_2_, NO_2_, and CO, emphasizing an association with the dosage, length of exposure (lag), or cumulative effects, also suggesting possible cut-off values specific for each pollutant [20]. A complex interaction, including an increased predisposal towards RSV infection, an intensified inflammatory response, and reduced local lung immunity, increases the risk of a more severe disease course, offering a plausible explanation of the role of ambient pollutants [16,21,22]. Depending on the study setting, population enrolled, or exposure lag time, the results may vary, but the significance of ambient pollution for respiratory infections is recognizable; however, due to limited data on this topic, broad research is needed. Moreover, air pollution data might be used both in models for the prediction of lower respiratory tract infections, which might ameliorate the management of healthcare systems, and in employing interventions addressed to communities, such as advice of a reduction in outdoor activities [15]. For those purposes, we aimed to verify if higher air pollution concentrations at a particular day influence the number of hospital admissions. This study analyzed a 10-year period of pediatric hospitalizations due to RSV LRTI in Poland, a country with a population of 38 million, in order to establish an association between air pollution and the number of hospital admitted RSV cases. It should be emphasized that Poland is characterized by high concentrations of air pollutants compared to other European Union countries (especially in the case of PM_10_ and PM_2.5_) [23], which increases the risk of respiratory problems [24], also among children [25].

## 2. Materials and Methods

### 2.1. Study Area and Participants

Poland covers an area of 312,700 km^2^ (120,700 mi^2^) and is inhabited by approximately 38 million people; about 20% of the population is under 18 years old. This study included children hospitalized due to RSV lower respiratory tract infections in 10 consecutive seasons (2010–2019). Data on hospitalizations were obtained thanks to the courtesy of the Polish National Health Fund (NFZ) and retrieved from the database of the NFZ, which is a public organization responsible for all public health services related to, inter alia, hospital treatment and its financing. In fact, the NFZ possesses data on the vast majority of hospitalizations within the Polish healthcare system model, where public hospitalizations play a major role in pediatric healthcare, with only a single number of commercial pediatric wards that provide pediatric hospital services, in a marginal proportion of cases. Patients were enrolled based on the final diagnosis, in accordance with the 10th edition of the International Statistical Classification of Diseases and Related Health Problems (ICD-10), which included: J12.1 pneumonia caused by RSV, J20.5 bronchitis caused by RSV, J21.0 bronchiolitis caused by RSV. Patients’ anonymized data were provided on a daily basis from newly diagnosed cases (the first day of hospitalization due to one of the above final diagnoses coded with ICD-10) and reported in the following age groups: 1–5 months old, 6–11 months old, 24–59 months old, 5–9 years old, 10–14 years old, 15–17 years old. Each case (event) was related to the community code of the service provider. Laboratory confirmation of the etiological factor was performed at the discretion of each service provider (hospital or hospital ward). Cases incorrectly classified as caused by RSV might have occurred; however, according to the Polish public healthcare system policy, it is expected that each service provider reports the final diagnoses of patients to the NFZ, which in turn, upon those diagnoses, reimburses the hospital treatment costs, in which the costs vary upon the disease and length of stay. In order to omit any risks of misclassification or fraudulent practices, the payer (the NFZ) holds and executes its right for medical documentation control and is authorized to impose a contractual penalty. Conversely, there is a risk of underestimation of RSV-caused hospitalizations, which might be reflected in an increasing number of diagnosed cases. This is most likely attributable to the improving access to microbiological diagnostics, including both rapid antigen tests and molecular methods, which have definitely become more available. Moreover, as in the case of other infectious diseases, significant interhospital divergencies are observed in terms of the access to microbiological diagnostics, frequency of their use, local guidelines/protocols or practices, and varied awareness of the RSV presence, and an important underestimation of RSV cases might be expected in certain hospitals. Finally, sites with the highest occurrences were analyzed; we empirically established the minimal number of events during the whole study period required for the model.

RSV exhibits a significant seasonality, and in order to estimate the influence of air pollutants, the division between low-risk (warm) and high-risk (cold) RSV seasons was established. The latter season occurs in Poland from October to April, which results in, among others, the need for the implementation of such prophylactic measures as passive immunoprophylaxis in high-risk patient groups [26]. This approach is in line with other authors investigating the influence of air contamination on RSV [12,14,27].

### 2.2. Pollution Data

Data on the concentrations of the following air pollutants were recorded: particulate matter of an aerodynamic diameter less than or equal to 2.5 μm (PM_2.5_), particulate matter of an aerodynamic diameter less than or equal to 10 μm (PM_10_), and nitrogen dioxide (NO_2_). Readings of air pollutants’ concentrations were obtained from the State Environmental Monitoring (SEM) network run by the Chief Inspectorate of Environmental Protection. Any location which had at least one year of continuous measurement history of any of the aforementioned air pollutant concentrations was eligible for the study. Only readings from air quality monitoring stations located in the studied sites were used, and in cases where there were more than one station in a location, a mean was calculated and analyzed as the urban background.

We consider these measurements to be representative for the assessment of the patients’ overall exposure to pollutants, although they were assigned to the hospital locations, not the patients’ addresses. The latter would be impossible due to patient data anonymization, and differences among declared and actual address of residence, as well as futile due to the fact that, although patients are free to choose any hospital in the country, the vast majority of hospitals serve local communities, and the choice of a hospital far from one’s home is an uncommon practice in Poland.

All the data on air pollutant concentrations were collected from the automatic air quality monitoring stations operating within the SEM; the concentrations were available in the form of 1 h mean values. Daily mean levels were calculated from the 1 h mean concentrations and then included in the statistical model. The list of the measuring stations is available at http://powietrze.gios.gov.pl/pjp/maps/measuringstation, accessed on 19 March 2021.

### 2.3. Statistical Analysis

We investigated the association between the health outcome (RSV pediatric hospitalizations based upon daily data collected by the National Health Fund from the period 2010–2019) and the air pollutant (PM_2.5_, PM_10_, and NO_2_) concentrations. The models included the daily pollutant concentrations, as well as the seasonal and annual periodicity (without meteorological factors), in order to identify the interrelationships with a full interaction scheme between the concentrations and the incidence of RSV. Initial analysis of the autocorrelation function (ACF) and partial autocorrelation function (PACF) was performed. To examine the short-term impact of air pollutants on RSV, we identified optimal general regression models for each agglomeration. After the analysis of the data quality and the total number of hospitalized cases, the 2012–2019 period was chosen, and data for the Warsaw, Krakow, Gdansk, Lodz, Wroclaw, Szczecin, and Walbrzych agglomerations were included in the final analysis. The daily data from the particular areas were collected, the models were created, and a generalized metaregression model was prepared in order to recapitulate the results.

### 2.4. General Regression Models (GRM)

To determine and characterize the statistically significant effects of single variables along with their interactions on the investigated health outcomes, we identified statistical models belonging to the family of GRM. GRM represent a group of models broadly applicable in the evaluation of complex experimental systems consisting of qualitative and quantitative data expressed on various scales. An important advantage of general linear models is their ability to describe nonlinear relationships between variables due to the application of appropriate transformations of predictive factors and the use of substitution of Z-standardized variable methods. As a result, complex equations describing nonlinear functions are transformed to simpler ones of a linear character. The GRM methodology, in contrast to the corresponding methods, includes the effects of interactions between two or more variables [28]. In our study, to ensure the simplicity of the model, we restricted the interactions to two variables only. In the first step of the multistep process of model building, the GRM model was identified, and the effects of single variables on a dependent variable were determined. In the case of GRM, a lack of an event is equivalent to a lack of data and reduces the model’s quality; thus, only locations where the number of events (i.e., days with hospitalization) allowed for the construction of a reliable model were included. Step 2 included the identification of models with the interactions. Pareto graphs were drawn in each step of the model identification in order to show the strength of the effects of the single variables and their interactions. Step 3 consisted of the metaregression models prepared for both univariate and multivariate analyses and was designed to briefly sum up the results. It needs to be emphasized that the metaregression model might be influenced by other site-specific factors (meteorological, for example) and is the recapitulation of the results only at the small expense of precision. Only statistically significant (*p* ≤ 0.05) variables were shown.

In each initial model, the health outcome represented the dependent variable. Independent variables were as follows: year, season (summer season, from May to September; cool season, from October to April, which corresponds to the RSV high-risk season), and concentrations of air pollutants. To identify the optimal (final) GRM model, a forward stepwise approach was applied. Models were evaluated using the adjusted coefficient of determination (R2):

(1) $$R^{2}=1-\frac{n-1}{n-k-1}\left(1-\frac{\sum_{t=1}^{n}{\left({\hat{x}}_{t}-\bar{x}\right)}^{2}}{\sum_{t=1}^{n}{\left(x_{t}-\bar{x}\right)}^{2}}\right)$$

where:

*x_t_*—value of variable X at time or period *t*;

${\hat{x}}_{t}$—theoretical value of variable X at time or period *t*;

$\bar{x}$—mean value of variable X in a time series of *n* observations;

*n*—number of observations;

*k*—number of explanatory variables.

Data pre-processing and calculations were performed with the use of Statistica version 13 (TIBCO Software Inc., Palo Alto, CA, USA) and dedicated EDM Eco Data Miner version 1.09.

## 3. Results

**Figure 1:**
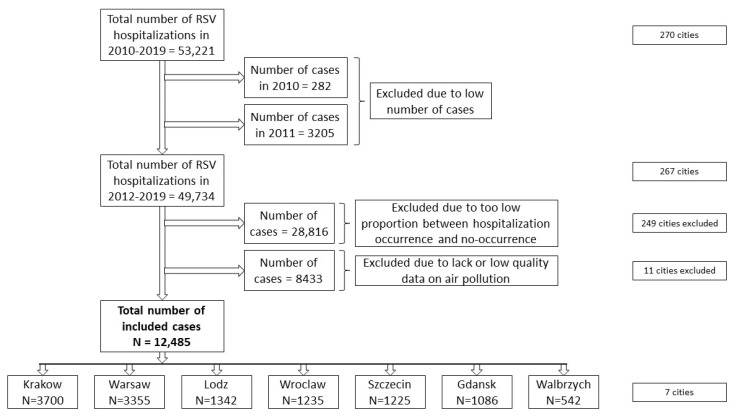
A flow chart of the RSV hospitalizations included in the study.

**Figure 2:**
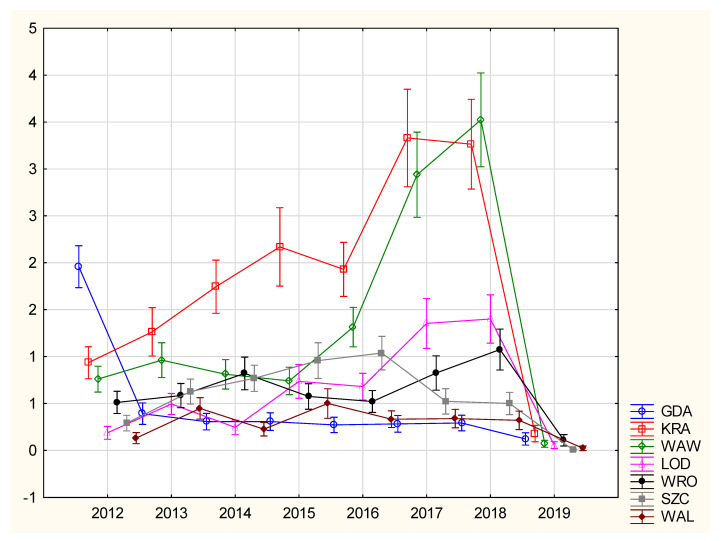
Annual distribution of mean number of RSV cases (per day) in the period included in the analysis. The vertical bars denote 0.95 confidence intervals. Abbreviations for the cities: GDA—Gdansk, WAW—Warsaw, KRA—Krakow, WRO—Wroclaw, LOD—Lodz, SZC—Szczecin, WAL—Walbrzych.

During the analyzed period of 2010–2019, a total number of 53,221 RSV hospitalizations took place (a total of 48,486 separate records based upon age group and a day during the studied period were retrieved from the NFZ database) in 270 cities (Figure 1). The RSV case distribution exhibited annual, quarterly, and seasonal changes (Figure 2 and Supplementary Materials Figure S1). Due to the low data quality, which mainly resulted from the very low number of diagnosed cases, the years 2010 and 2011 were excluded from further analysis. Since the low proportion between hospitalization occurrence and non-occurrence would affect the statistical models (Step 1 of the statistical analysis), 28,816 hospitalizations were excluded; a further 8433 hospitalizations were excluded due to a lack of or a low quality of air pollution measurements. Finally, 12,485 hospitalizations in 7 cities were included in this study (Figure 1). The air pollution data in the analyzed sites and period showed a high quality, and only in one site (Walbrzych) were PM_10_ measurements lacking for 2010 and 2011; however, those years were not included in the final analysis for the reasons mentioned above (Table 1 and Supplementary Materials Figure S2). The distribution of the autocorrelation function for locations was similar for each of the analyzed pollutant concentrations, separately for each city, similar to the partial autocorrelation function (Supplementary Materials Figure S3). The autocorrelation function shows slow changes, while for the partial autocorrelation, an influence of the trend (the first lag of PACF) was seen. There was also a weak, yet significant, correlation between the pollutants (Supplementary Materials Table S1).

Among the seven sites with the highest number of RSV cases in the analyzed period, both univariate and multivariate models showed that a significant part of the RSV hospitalizations may be attributable to air pollution: the coefficient of determination (R^2^) varied between 0.049 (Walbrzych) and 0.31 (Gdansk) in the univariate models, and between 0.084 (Walbrzych) and 0.31 (Gdansk) in the multivariate models; in each of the analyzed sites (except for Gdansk), the R^2^ was higher in the multivariate model (Table 2). In the metaregression analysis, the R^2^ was also higher in the multivariate models than in the univariate models (0.314 vs. 0.23, respectively) (Table 2).

In the univariate model, the most significant effect was observed for PM_2.5_ and NO_2_, which showed a significant association with RSV hospitalizations in five out of seven sites, while PM_10_ showed a significant association in four sites, and the metaregression revealed statistical significance for each of the analyzed parameters.

**Figure 3:**
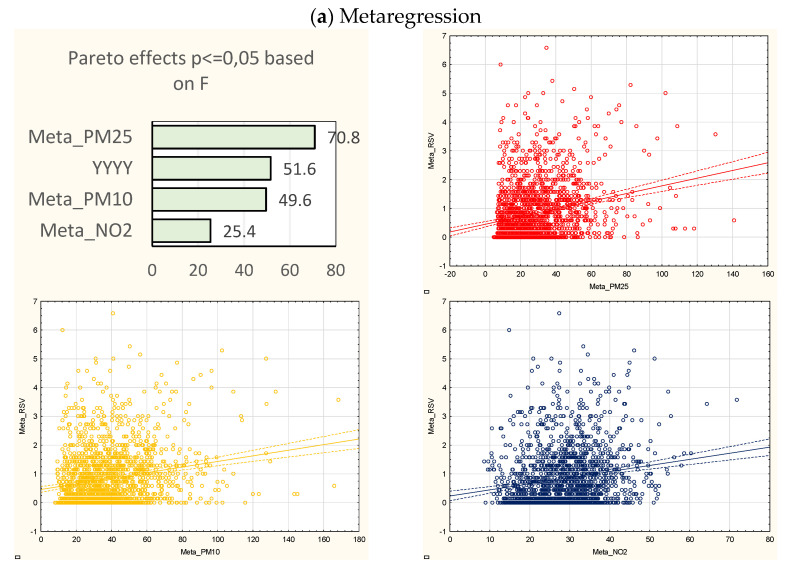

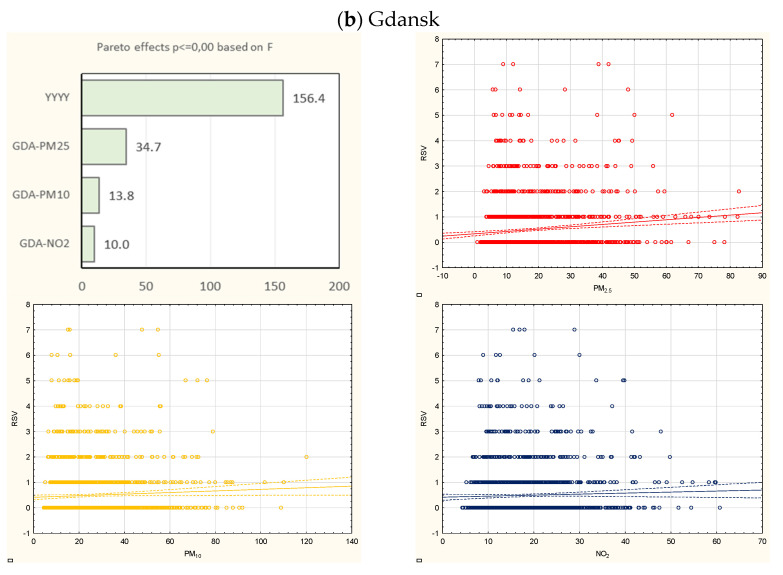
Pareto charts of the effect power (based on the F statistics) and corresponding scatterplots of the air pollutant concentrations and RSV hospitalizations (sequentially: PM_2.5_, PM_10_, NO_2_) during the RSV high-risk seasons (2012–2019) in metaregression, and on the example of Gdansk (see also Supplementary Materials Figure S4, where full data on each of the sites are presented). Abbreviations: YYYY- years of the study; Meta_ or GDA_PM_2.5_-PM_2.5_ in metaregression or Gdansk, respectively; Meta_ or GDA_PM_10_-PM_10_ in metaregression or Gdansk, respectively; Meta_ or GDA_NO_2_-NO_2_ in metaregression or Gdansk, respectively.

A 10 μg/m^3^ increase in the PM_2.5_ concentration was associated with the mean increase in RSV hospitalizations of 0.087 to 0.16, depending on the location, whereas the metaregression model showed an average increase of 0.134. A 10 μg/m^3^ PM_10_ increase corresponded to a mean increase in RSV hospitalizations of 0.031 to 0.087 (metaregression average = 0.097), while a 10 μg/m^3^ increase in NO_2_ concentrations resulted in a mean increase of 0.04 to 0.29, and this was associated with an average increase of 0.21 in the metaregression (Figure 3).

Among the specific study locations, each of the analyzed pollutants played a significant role in Gdansk (R^2^ = 0.31), Krakow (R^2^ = 0.17), and Wroclaw (R^2^ = 0.092). A 10 μg/m^3^ increase in PM_2.5_ corresponded to a 0.092, 0.132, and 0.088 increase in RSV hospitalizations, respectively, while a 10 μg/m^3^ increase in the PM_10_ levels was associated with 0.031, 0.087, and 0.06 increases in the number of cases, respectively. The NO_2_ 10 μg/m^3^ increase was related to a 0.04, 0.29, and 0.104 increase in the hospitalizations, respectively. NO_2_ was the only air pollutant significantly associated with increased hospitalization rates in Szczecin (R^2^ = 0.1 for the model) and Walbrzych (R^2^ = 0.05), and an increase of 10 μg/m^3^ was related to a 0.16 and 0.093 increase in the events, respectively. NO_2_ remained insignificant in Warsaw (R^2^ = 0.25), while a 10 μg/m^3^ increase in the PM_2.5_ or PM_10_ concentration corresponded to a 0.16 and 0.146 increase in hospitalizations, respectively. PM_2.5_ was the only significant air pollutant in Lodz (R^2^ = 0.15), and its 10 μg/m^3^ increase corresponded to a 0.087 increase in the number of cases (Figure 3).

**Figure 4:**
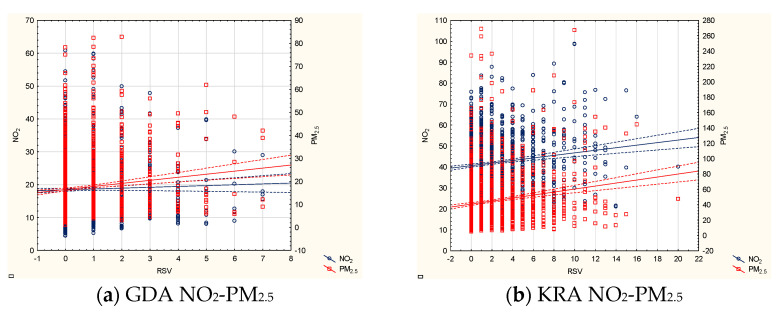

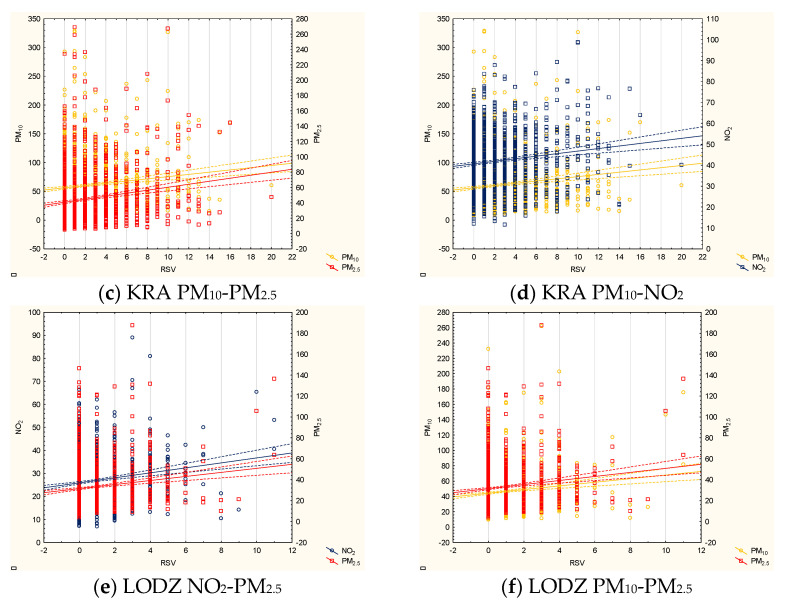
Scatterplots of the interactions between the air pollutant concentrations and RSV hospitalizations during the RSV high-risk season (2012–2019) in the study sites (only significant results are shown): (**a**) Gdansk: NO_2_ and PM_2.5_ against RSV hospitalizations, (**b**) Krakow: NO_2_ and PM_2.5_ against RSV hospitalizations, (**c**) Krakow: PM_10_ and PM_2.5_ against RSV hospitalizations, (**d**) Krakow: PM_10_ and NO_2_ against RSV hospitalizations, (**e**) Lodz: NO_2_ and PM_2.5_ against RSV hospitalizations, (**f**) Lodz: PM_10_ and PM_2.5_ against RSV hospitalizations.

In the multivariate model, PM_2.5_ presented the most significant effect, as the sum of its effects reached 12, which was followed by PM_10_ and NO_2_ (both effects summed up to 10, Table 2). Single air pollutant concentrations alone showed significant associations: PM_2.5_ was related to the number of hospitalizations in two sites (Warsaw and Wroclaw), PM_10_ alone was a significant factor in three locations (Gdansk, Warsaw, Wroclaw), and NO_2_ was also significant in three sites (Wroclaw, Szczecin, Walbrzych). PM_2.5_ interacted with NO_2_ in three locations, along with PM_10_ in two locations, while PM_10_ and NO_2_ interacted in one location (Figure 4).

**Figure 5:**
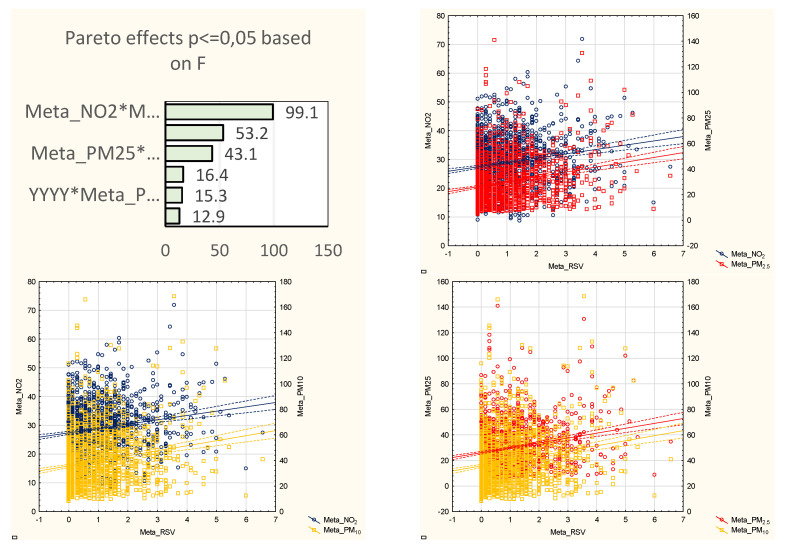
Pareto chart of the effect power (based on the F statistics) in the metaregression and scatterplots of the interactions between the air pollutant concentrations and RSV hos pitalizations during the RSV high-risk season (2012–2019) in the metaregression. Abbreviations: Meta_PM_2.5_-PM_2.5_ in metaregression; Meta_PM_10_-PM_10_ in metaregression; Meta_NO_2_-NO_2_ in metaregression; Meta_NO_2_*Meta_PM_2.5_- interaction between NO_2_ and PM_2.5_; Meta_NO_2_*Meta_PM_10_- interaction between NO_2_ and PM_10_; Meta_PM_2.5_*Meta_PM_10_- interaction between PM_2.5_ and PM_10_; YYYY*Meta_PM_2.5_- interaction of the years of the study and PM_2.5_; YYYY*Meta_PM_10_- interaction of the years of the study and PM_10_; YYYY*Meta_NO_2_- interaction of the years of the study and NO_2_.

Vast multifarious interactions were observed in Krakow (R_2_ = 0.2), where PM_2.5_ interacted with NO_2_ and separately with PM_10_, and an interaction between PM_10_ and NO_2_ was also observed. The PM_2.5_–NO_2_ and PM_2.5_–PM_10_ interactions were significantly associated with the number of cases in Lodz (R^2^ = 0.16). Additionally, PM_2.5_–NO_2_ was a significant factor in Gdansk (R^2^ = 0.31), where PM_10_ was independently associated with the number of cases. Interestingly, irrespectively of the multivariate model, PM_2.5_ and PM_10_ were independent factors in Warsaw (R^2^ = 0.3) and Wroclaw (R^2^ = 0.15), and in Wroclaw, NO_2_ was also an independent factor. NO_2_ in the multivariate model was independently related to RSV hospitalizations in Szczecin (R^2^ = 0.13) and Walbrzych (R^2^ = 0.08). The metaregression showed statistically significant interactions between each of the pollutants as well as between the pollutants and the year of the study; the final model included interactions between PM_2.5_ and NO_2_, PM_2.5_ and PM_10_, and PM_10_ and NO_2_, as well as between PM_2.5_ and year, PM_10_ and year, and NO_2_ and year (Figure 5). The summary effect was similar but slightly stronger than the strongest effect found in the city-by-city analysis (R^2^ = 0.314 compared to 0.31).

## 4. Discussion

This study shows a significant contribution of air pollutants to RSV hospitalizations and underlines the complexity of interactions. Depending on the model used, RSV hospitalizations can be explained at 23% (in the univariate model) or 31.4% (in the multivariate model) by models that include three air pollutants: PM_2.5_, PM_10_, and NO_2_. Although we focused on hospitalized cases only, the results are similar to those obtained by Qing Ye in a large-scale outpatient study, which enrolled 3650 pediatric RSV patients [20]. Qing Ye reported a correlation between PM_2.5_, PM_10_, and NO_2_, as well as SO_2_ and CO, and the RSV infection rate (r = 0.446, 0.397, 0.365, 0.389, and 0.532, respectively) [20]. The complexity of interactions is reflected by the different influences of the lag and the cumulative effect in various pollutants. In the case of PM_2.5_, its influence decreased with the duration of the lag, similar to CO, but PM_10_ started to increase the RSV positivity rate after a 3-day lag, while the greatest risk increase in the case of NO_2_ and SO_2_ was observed at a 3-day lag [20]. Moreover, while there was no cumulative effect of PM_2.5_, PM_10_, NO_2_, or SO_2_, one was observed for CO (with the peak at the CO level of 1.5 mg/m^3^) [20]. The choice of air pollutant concentrations on the day of hospital admission might seem controversial, but this simplification of the relationship combines various pollutants’ effects and verifies the basics for the creation of easy-to-introduce practices in the communities, e.g., air pollution alerts encouraging people to decrease outdoor activities [15]. While complex interactions between the pollutants were observed in our study, the influence of each particular pollutant should also be known. The effect of PM_10_ found in our study is in line with other authors. Short-term (as well as medium-term) exposure to PM_10_ was recently associated with an increased risk of RSV hospitalization by Carugno, who investigated 2814 infants hospitalized due to RSV bronchiolitis [14]; our results also show the important role of PM_10_ in both univariate and multivariate models, and a 10 μg/m^3^ increase in PM_10_ corresponded to a 0.097 increase in RSV hospitalizations in our series of patients. The study by Carugno also addressed the question of different lags of exposure, including single days before hospitalization (0 to 30), the average (lags 0–1 to 0–30), and up to 4 weeks before hospitalization [14]. A 0-day lag, which was used in our calculations, showed a 6% increase in the hospitalization risk, while a 1-day lag was associated with a 7% increase; then, the risk started to slowly decrease until lag 12, when no increased risk was observed [14]. Higher risks were seen regarding the average exposures (from 1.08 for 0–1 lag to 1.15 for 0–13 lag), while weekly lags showed an increased risk by 6% and 7% for weeks 1 and 2 prior to hospitalization [14]. Current exposure to PM_10_ increased the risk of RSV hospitalization in children by 1.21% in a study by Fukuda, who analyzed the effects of PM_10_ on viral respiratory hospitalizations [10]. The highest risk increase, however, was observed at a longer lag (7–14 days), followed by a 1–6-day lag (3.16%, and 2.87%, respectively [10]). We found that PM_10_ might also have an immediate effect—we only analyzed the concentrations on the day of hospital admission. This is in contrast with Vandini, who included 327 infants under 2 years of age consulted at an emergency department due to RSV infection and proved a correlation between the RSV activity and PM_10_ a week before the ED visit (r = 0.34), but not at lag 0 [12]. A slightly lower correlation with PM_2.5_ was also observed (r = 0.26, p within the statistical significance range), yet the authors assumed the coefficient should exceed 0.3 to be considered significant [12]. Similarly, Yitshak-Sade analyzed 4069 bronchiolitis hospitalizations (not restricted to RSV bronchiolitis) and found that an increase in both PM_10_ and PM_2.5_, as well as NO_2_, was related to an increased risk of bronchiolitis (odds ratio, OR of 1.06, 1.04, and 1.36, respectively), although there was no association with pollutants’ IQR at 0–4 days prior to hospitalization [13].

We found that PM_2.5_ exposure (without any lag) was significantly associated with RSV hospitalizations in our models (uni- and multivariate), while previous studies showed the higher risk to be related to longer exposure periods. A cumulative effect of PM_2.5_ concentrations was observed by Horne, who analyzed 130,295 children hospitalized due to acute LRTI (including 35,774 and 2236 RSV diagnosed cases aged 0–2 and 3–17 years old, respectively) [16]. The risk of LRTI increased within 1 week and reached the highest values after 3 weeks of exposure, with a total cumulative OR (28 days) of 1.15 per 10 μg/m^3^ increase in PM_2.5_ concentrations [16]. In the case of laboratory-confirmed RSV cases in children, the highest OR was observed in week 2 (ages 0–2) or 3 (ages 3–17) [16]. Short- and long-term exposures to PM_2.5_ were assessed by Karr in a case–control investigation of 2604 cases of bronchiolitis matched with 23,354 controls; the analyzed lags included 7, 30, and 60 days prior to the bronchiolitis episode, and lifetime exposure, regardless of its length [17]. Interestingly, a higher risk was seen with increasing exposure time. The adjusted odds ratio for RSV bronchiolitis hospitalization reached 1.14 (95%CI: 0.88–1.46) per 10 μg/m^3^ increase in lifetime PM_2.5_ exposure [17].

NO_2_ mentioned above, which was associated with more frequent RSV infections in an outpatient setting in the study by Qing Ye [20], was also shown to be positively associated with the number of consultations and hospitalizations based on data from emergency departments of 34 hospitals in Paris, which included 50,857 bronchiolitis consultations and 16,588 hospital admissions (in children under 3 years of age) [11]. We found an immediate effect of NO_2_ exposure on RSV hospitalizations, and the metaregression analysis showed it was associated with an average increase of 0.21 in hospitalizations. On the contrary, Segala showed that the NO_2_ effect is statistically significant only at a longer window of exposure (0–4-day lag, but not 0–1-day lag) and results in increased odds of both consultations (3% increase) and hospitalizations (4% increase) [11]. Interestingly, the same cumulative effect caused the statistical significance in the case of PM_10_ [11].

The vast majority of studies have been performed based on hospitals, due to the availability of laboratory diagnostics, including rapid antigen tests, serological studies, and molecular methods used to confirm the RSV etiology. In this study, we also chose a hospital-based setting, for three main reasons: (1) laboratory confirmation, which was not verified in each case, but we assume that the diagnoses (coded with ICD-10) truly reflect the etiological factor, since the legal regulations on public financing of hospital treatment demand the final diagnosis (coded with ICD-10) to be proved, or, otherwise, the payer may execute financial penalties; (2) hospital treatment is the most important cost driver in healthcare systems, and possible profits of improved air quality may be expected mainly in this sector; (3) the exact pathomechanism of the activity of pollutants regarding RSV morbidity has not been established, and a mixed effect on the susceptibility to infection and its worse course (resulting in hospitalization) is suspected; thus, the effects of air pollution would be more apparent in hospitalized cases.

In order to understand the differences between the lag and cumulative and immediate effects (found in our study) of air pollutants on RSV hospitalizations, in-depth knowledge on the underlying pathomechanisms and clinical course of the disease is needed. A possible explanation of the impact of air pollution has been the subject of many investigations, yet scarce definite answers have been offered. PM_10_ has been suggested to alter the inflammatory response regulated by alveolar macrophages and facilitate the spread of infection, leading to a worse clinical course [29], while NO_2_ impairs the epithelium functions, ozone increases epithelial cells’ permeability, and particulate matter (PM) induces oxidative stress, thus affecting both specific and non-specific lung immunity [22]. A putative complex mechanism of PM_2.5_, for example, is explained by an increased susceptibility in the population, due to the impaired local immunity, which, along with a prolonged disease course, leads to an increased exposure to highly infectious diseases. This causes a more severe course, as the oxidative stress and inflammation are augmented by air pollution [16]. Additionally, a synergistic action of RSV activity and exposure to PM_2.5_ has been described and needs to be taken into consideration [30]. Of note, certain clinical studies suggest the presence of a cut-off value, above which a negative effect of a pollutant is pronounced more clearly; for example, PM_2.5_ starts to influence the RSV incidence at a concentration of over 150 μg/m^3^ [20]. The question of differences between polluted and clear locations has already been raised. While the majority of studies focused on urban or industrial areas (Paris, Rome, Lombardy, Christchurch, Santiago de Chile) [10,11,14,18,30], studies performed in areas with substantially variable air pollution [16] or low air pollutant concentrations also provide similar results [17]. The choice of sites in this study was based upon the total number of diagnosed RSV cases, not upon the air pollution levels. As a result, we analyzed both highly polluted sites and less-polluted areas.

Certainly, there are evident discrepancies in the published data, and some studies did not confirm a relationship between the particular air pollutants and RSV; for example, an analysis of 1670 RSV hospitalizations in children under 36 months of age matched with 6680 controls showed an increased risk of hospitalization in relation to higher annual ozone levels (OR = 1.03 per 1 parts per billion/ppb increase), but not with annual PM_2.5_ or NO_2_, yet the authors concluded that these figures might be underestimated due to the annual values that were used for the calculations [19]. On the other hand, an analysis of 266 infants with RSV bronchiolitis showed lower and higher PM_10_, PM_2.5_, benzene, NO, and SO_2_ concentrations in the months with a high RSV activity, although the only independent predictor of the RSV incidence was the benzene concentration [18]. In order to omit the strong influence of seasonality, the models finally included only cases hospitalized within the high-RSV risk season.

There are certain strengths as well as limitations to this study. The huge patient cohort which reflects all the pediatric hospitalizations in a large European country guarantees the quality of the data. However, the flow of the patients needs to be discussed first. Although the initial number of eligible patients exceeded 53,000, finally, 12,485 hospitalizations were included. The most significant reason for the exclusion was the too low proportion between the events and lack of events (hospitalizations and a lack of hospitalizations on particular days), which is crucial for the construction of GRM; in order to ensure a high quality of the models, data that might raise doubts were excluded. Secondly, the lack of RSV laboratory confirmation, or, rather, the lack of its verification, is questionable, although the same methodology has been applied by other authors who based their investigation on huge hospital databases [14]. In fact, the effect of pollution may be rather underestimated than overestimated, since a stronger relationship between air pollution and RSV bronchiolitis, rather than unspecified bronchiolitis, was observed [17]. However, only minor differences were noted between clinically diagnosed and laboratory-confirmed RSV cases, with a tendency to a more pronounced effect in the latter cases [16]. Thirdly, a correction for air quality measurements needs to be taken into account; we used readings from the hospital area (as discussed in the methods section), which does not necessarily directly correspond to the patients’ exposure at home. Additionally, the measuring stations are located within a certain distance from the sites; however, other authors used readings from local capital cities or stations as distant as 20 km, and no distance-related obstacles have been reported [14,17]. Finally, we did not verify the correlation between the meteorological conditions and RSV hospitalizations suggested by other studies [18,31,32,33], since our study analyzed diverse sites located in practically every region of the country. Furthermore, the weather itself influences air pollution, and the actions that can be undertaken would focus on the reduction in air contamination.

In conclusion, air pollutants play a significant role in pediatric hospitalizations due to RSV lower respiratory tract infections. The inclusion of PM_2.5_, PM_10_, and NO_2_ in GRM explains a significant part of RSV hospitalizations. The pollutants act alone and interact in a varied manner. The interactions between the pollutants and the combined effects cannot be underestimated. Encouraging citizens to reduce outdoor activities on the days with high air pollution levels seems to be justified, while actions reducing air pollution might decrease the costs of hospital healthcare.

## Figures and Tables

**Table 1:** Mean air pollutant (NO_2_, PM_2.5_, and PM_10_) concentrations in different study sites (expressed in micro-g/m^3^).

Site:	Walbrzych			Wroclaw			Lodz			Krakow		
	NO_2_	PM_2.5_	PM_10_	NO_2_	PM_2.5_	PM_10_	NO_2_	PM_2.5_	PM_10_	NO_2_	PM_2.5_	PM_10_
2010	15.66	27.91		37.69	33.11	56.99	26.91	30.08	44.21	46.21	45.57	64.90
2011	17.52	21.76		43.06	31.52	48.80	25.90	27.75	37.38	45.31	44.59	64.77
2012	16.81	22.39	26.58	37.27	29.58	38.09	25.54	26.44	35.90	43.19	41.83	59.42
2013	14.35	23.81	33.27	44.04	29.09	36.31	25.41	26.48	39.52	39.98	35.35	50.20
2014	13.79	24.36	31.78	35.20	27.13	40.68	23.00	28.09	40.37	38.12	34.64	50.36
2015	15.02	18.91	27.22	31.94	25.38	33.58	24.81	24.57	37.24	40.93	35.09	51.28
2016	15.66	18.88	27.44	31.08	23.95	33.28	25.28	22.85	35.81	41.05	29.61	40.98
2017	15.21	21.39	27.06	28.62	22.01	30.18	24.33	25.87	38.29	41.07	32.84	42.90
2018	15.22	21.58	28.75	27.90	22.08	30.09	25.03	24.38	36.63	40.35	29.69	40.68
2019	13.11	15.36	23.09	26.16	17.15	25.36	21.46	20.17	31.96	39.02	24.82	34.83
Total	15.25	21.61	28.14	34.28	26.10	37.34	24.77	25.67	37.73	41.52	35.40	50.03
**Site:**	**Warsaw**			**Gdansk**			**Szczecin**					
	**NO_2_**	**PM_2.5_**	**PM_10_**	**NO_2_**	**PM_2.5_**	**PM_10_**	**NO_2_**	**PM_2.5_**	**PM_10_**			
2010	32.80	29.00	38.06	18.52	20.22	27.86	23.05	18.74	29.66			
2011	33.06	28.48	36.96	17.43	17.86	24.83	20.72	17.70	27.44			
2012	28.73	28.03	36.51	16.91	18.24	23.23	20.14	16.27	22.69			
2013	38.26	25.03	32.64	15.37	13.43	20.09	30.73	15.64	22.93			
2014	35.36	25.26	33.23	17.00	16.62	25.76	22.16	19.60	25.94			
2015	38.33	22.55	33.10	17.24	13.57	22.89	21.63	16.26	23.69			
2016	37.15	21.86	33.52	16.71	11.44	20.07	21.97	16.40	23.31			
2017	34.41	23.26	34.81	16.46	11.70	20.48	18.72	17.13	22.69			
2018	34.53	22.15	36.35	17.65	15.94	26.45	20.71	18.75	24.72			
2019	29.73	17.45	27.64	15.54	13.90	21.25	14.47	14.54	21.05			
Total	34.23	24.31	34.28	16.88	15.29	23.29	21.43	17.10	24.41			

**Table 2:** The results of the general regression models and the metaregression model in the univariate and multivariate analyses in the study sites (GDA—Gdansk, WAW—Warsaw, KRA—Krakow, WRO—Wroclaw, LOD—Lodz, SZC—Szczecin, WAL—Walbrzych). The subsequent rows show: the percentage of the RSV hospitalizations explained by the air pollution (R^2^) model with and without interactions, the degree of freedom for each study site, the number of statistically significant effects in the models with and without interactions (YYYY—year of the study), and statistically significant effects in the multivariate and univariate models according to the study site.

RSV									
	City code	GDA	WAW	KRA	WRO	LOD	SZC	WAL	
percentage of RSV hospitalizations explained by the air pollution (R2) model with interactions	Adjusted R^2^ (interactions)	30.5%	29.6%	20.4%	14.8%	16.2%	12.6%	8.4%	
		Metaregression							
		31.4%							
percentage of RSV hospitalizations explained by the air pollution (R2) model without interactions	Adjusted R^2^ (univariate)	31.4%	25.2%	17.3%	9.2%	14.7%	10.1%	4.9%	
		Metaregression							
		23%							
degree of freedom	Df = n − 1	3 634	1 698	1 897	1 678	1 686	1 691	1 262	Sum of the effects
Interactions	YYYY	1	3	3	4	1	3	2	17
	PM_10_	1	2	2	2	1	1	1	10
	PM_2.5_	1	2	3	2	2	1	1	12
	NO_2_	1	0	3	2	2	1	1	10
Univariate factors	YYYY	1	1	1	1	1	1	1	7
	PM_10_	1	1	1	1	0	0	0	4
	PM_2.5_	1	1	1	1	1	0	0	5
	NO_2_	1	0	1	1	0	1	1	5
	Interactions	YYYY	PM_2.5_	PM_2.5_*NO_2_	PM_2.5_	YYYY*NO_2_	NO_2_	NO_2_	
		NO_2_*PM_2.5_	PM_10_	PM_2.5_*PM_10_	PM_10_	NO_2_*PM_2.5_	YYYY	YYYY*PM_2.5_	
		PM_10_	YYYY	PM_10_*NO_2_	NO_2_	PM_2.5_*PM_10_	YYYY*PM_2.5_	YYYY*PM_10_	
			YYYY*PM_2.5_	YYYY	YYYY*NO_2_		YYYY*PM_10_		
			YYYY*PM_10_	YYYY*NO_2_	YYYY				
				YYYY*PM_2.5_	YYYY*PM_10_				
					YYYY*PM_2.5_				
	Univariate factors	YYYY	YYYY	YYYY	PM_2.5_	YYYY	YYYY	NO_2_	
		PM_2.5_	PM_2.5_	PM_2.5_	PM_10_	PM_2.5_	NO_2_	YYYY	
		PM_10_	PM_10_	PM_10_	YYYY				
		NO_2_		NO_2_	NO_2_				

## Data Availability

Data were obtained from the Polish National Health Fund (NFZ) and are available from the authors with the permission of the NFZ.

## Supplementary Materials

The following are available online at: https://www.mdpi.com/article/10.3390/jcm10153224/s1. Figure S1: The distribution of the RSV cases: (a) annual graph, (b) seasonal graph (cool versus warm season), (c) quarter graph, (d) seasonal and annual graphs combined with corresponding tables (table A—annual, B—seasonal, and C—annual and seasonal). The vertical bars denote 0.95 confidence intervals. The tables show mean and standard error and 95% confidence intervals. Figure S2. The annual boxplots of air pollutants’ (PM_2.5_, PM_10_, NO_2_) concentrations in the studied cities throughout the analysed period (2012–2019). Figure S3. The exemplary results of the city to city comparisons of autocorrelation function (ACF) and partial autocorrelation function (PACF) for Warsaw and Gdansk. Figure S4. The Pareto charts of the effects power (based on the F statistics) and corresponding scatterplots of the air pollutants concentrations and the RSV hospitalizations (sequentially: PM2.5, PM10, NO2) during the RSV high-risk seasons (2012–2019) in selected study sites. Table S1. A correlation between the air pollutants’ concentrations.
