# Supplementary material for: Air Pollutants’ Concentrations Are Associated with Increased Number of RSV Hospitalizations in Polish Children
Source: J Clin Med. 2021 Jul 22;10(15):3224. doi: 10.3390/jcm10153224 (PMC8348891; doi:10.3390/jcm10153224)
Supplement: Supplementary file 1 [file jcm-10-03224-s001.zip › Supplementary materials 1 revised.pdf]

Supplementary materials 1.

The distribution of the RSV cases: a) annual graph, b) seasonal graph (cool versus warm season), c) quarter graph, d) seasonal and annual graphs combined with corresponding tables (table A- annual, B- seasonal, and C- annual and seasonal). The vertical bars denote 0.95 confidence intervals. The tables show mean and standard error and 95% confidence intervals. Abbreviations for the cities: GDA- Gdansk, WAW- Warsaw, KRA-Krakow, WRO- Wroclaw, LOD- Lodz, SZC- Szczecin, WAL- Walbrzych

a) annual distribution

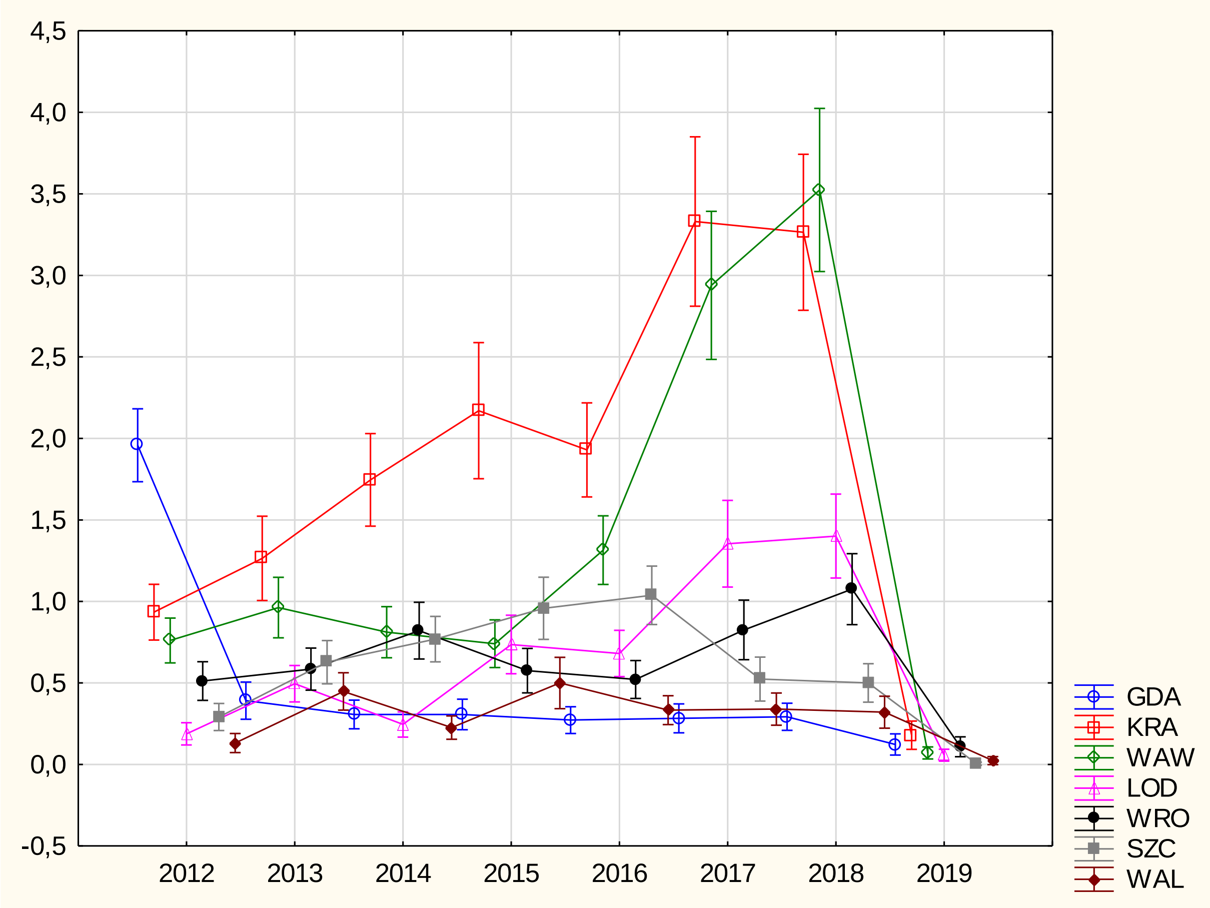

b) seasonal distribution

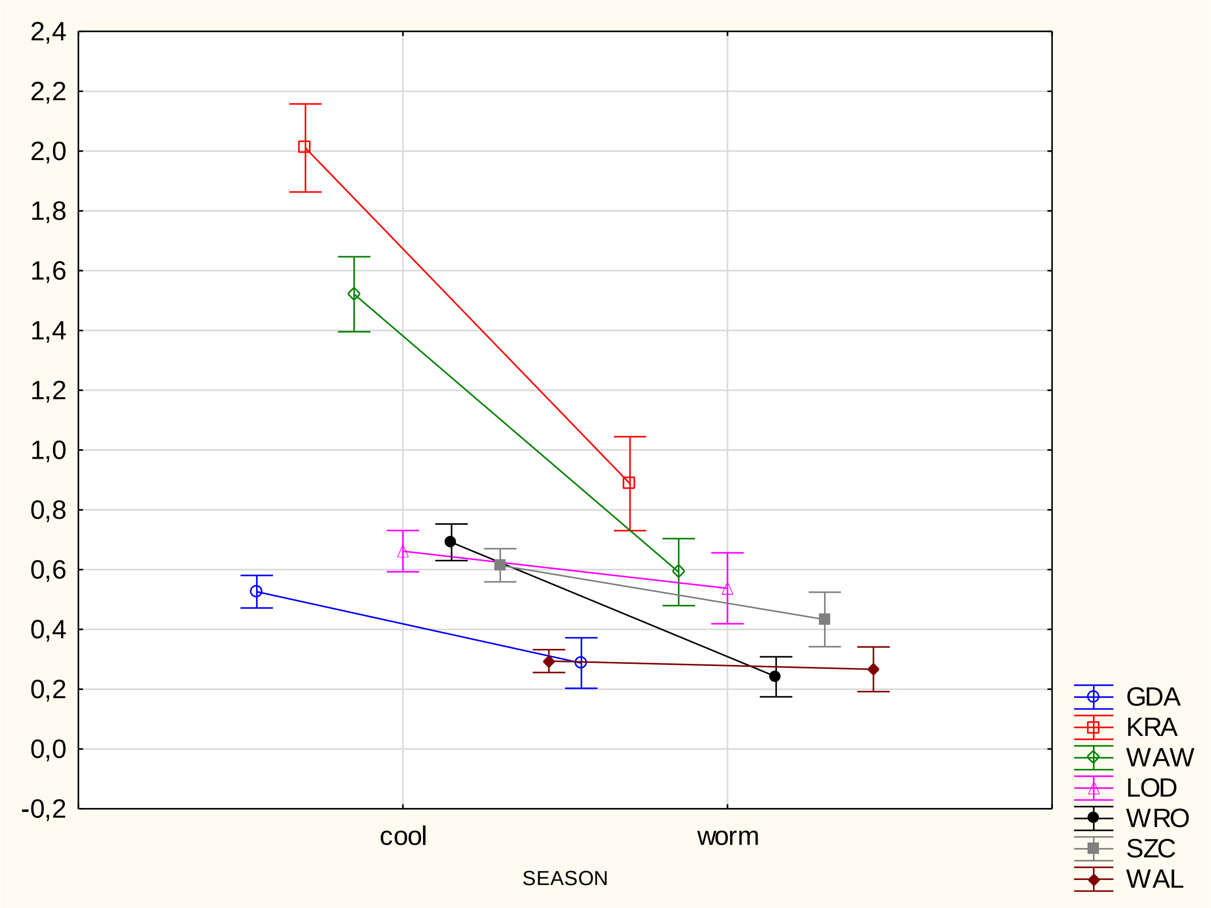

c) quarter distribution

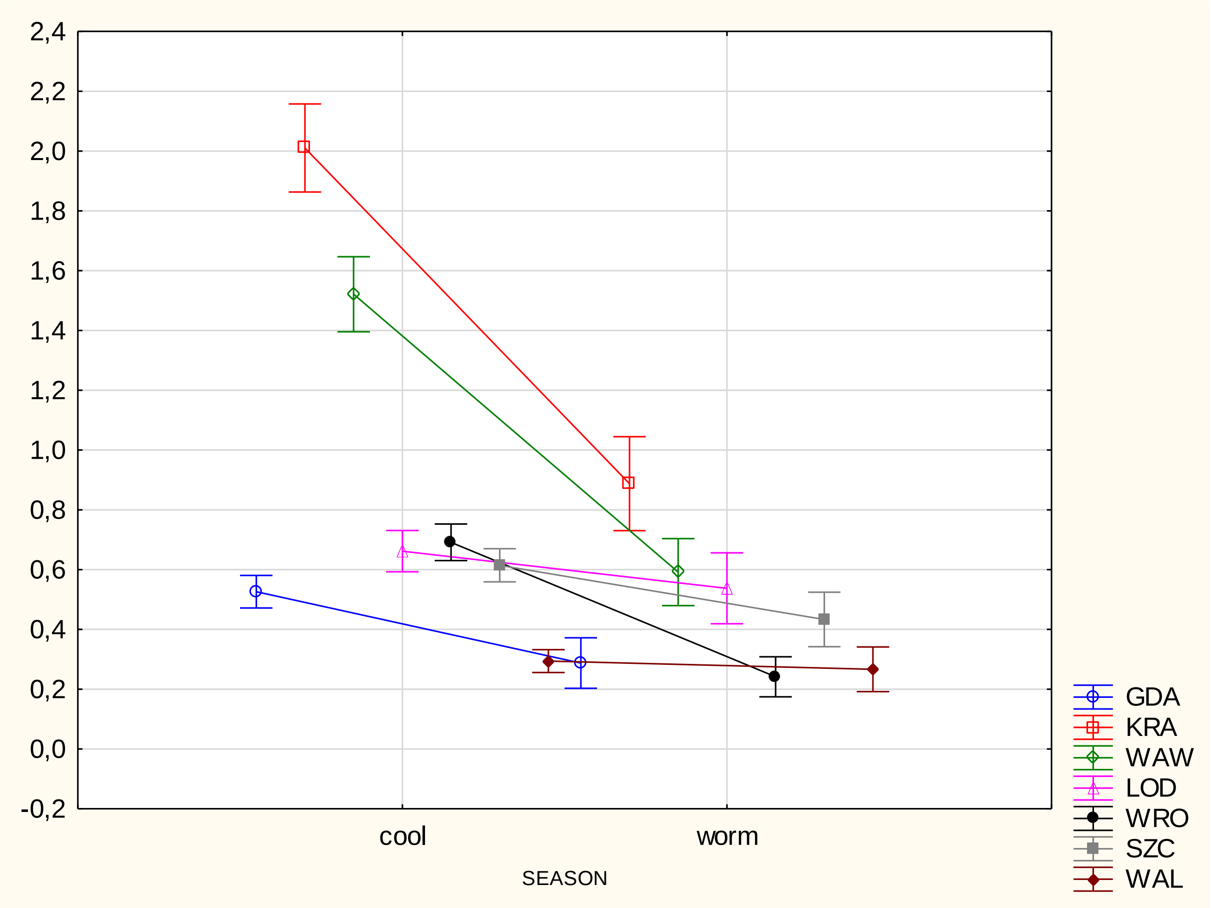

d) seasonal and annual distribution combined

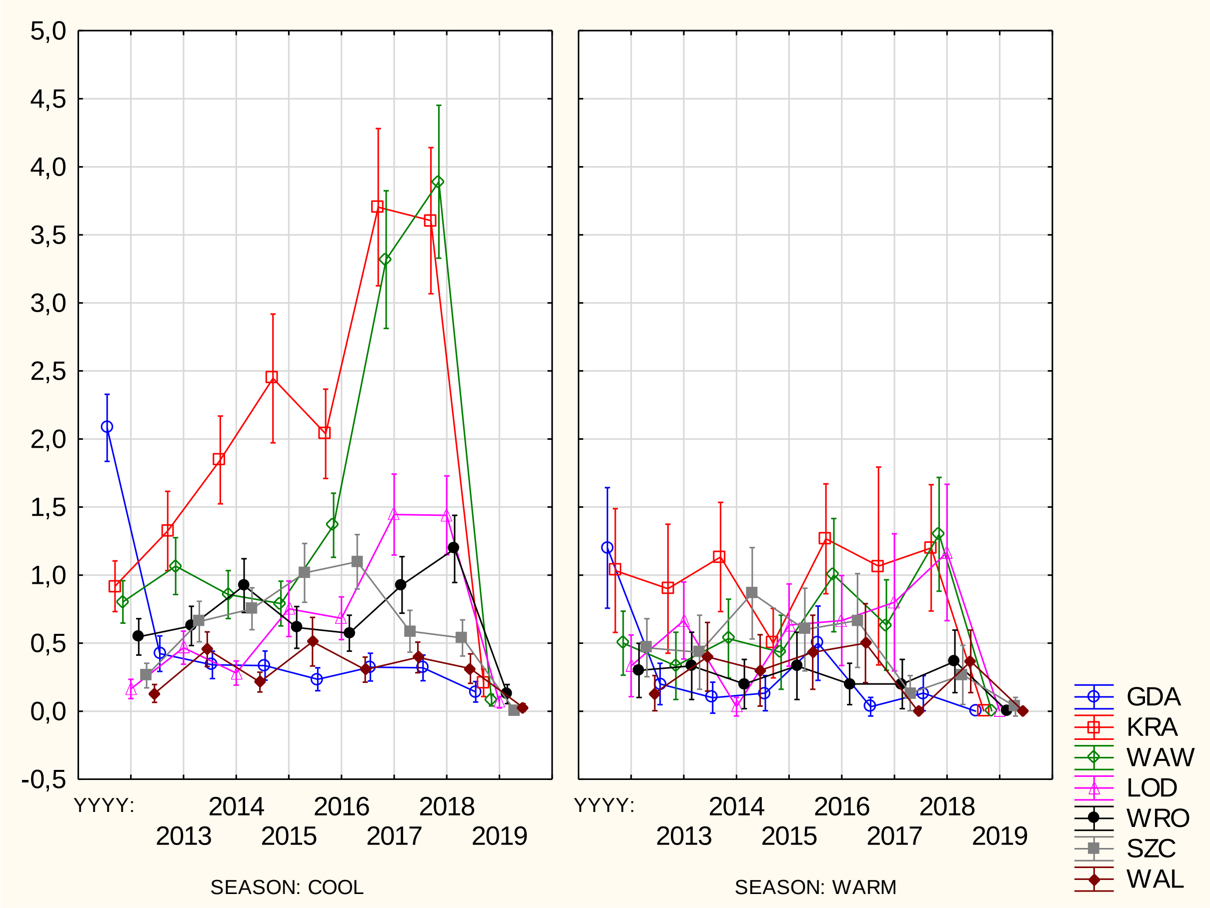

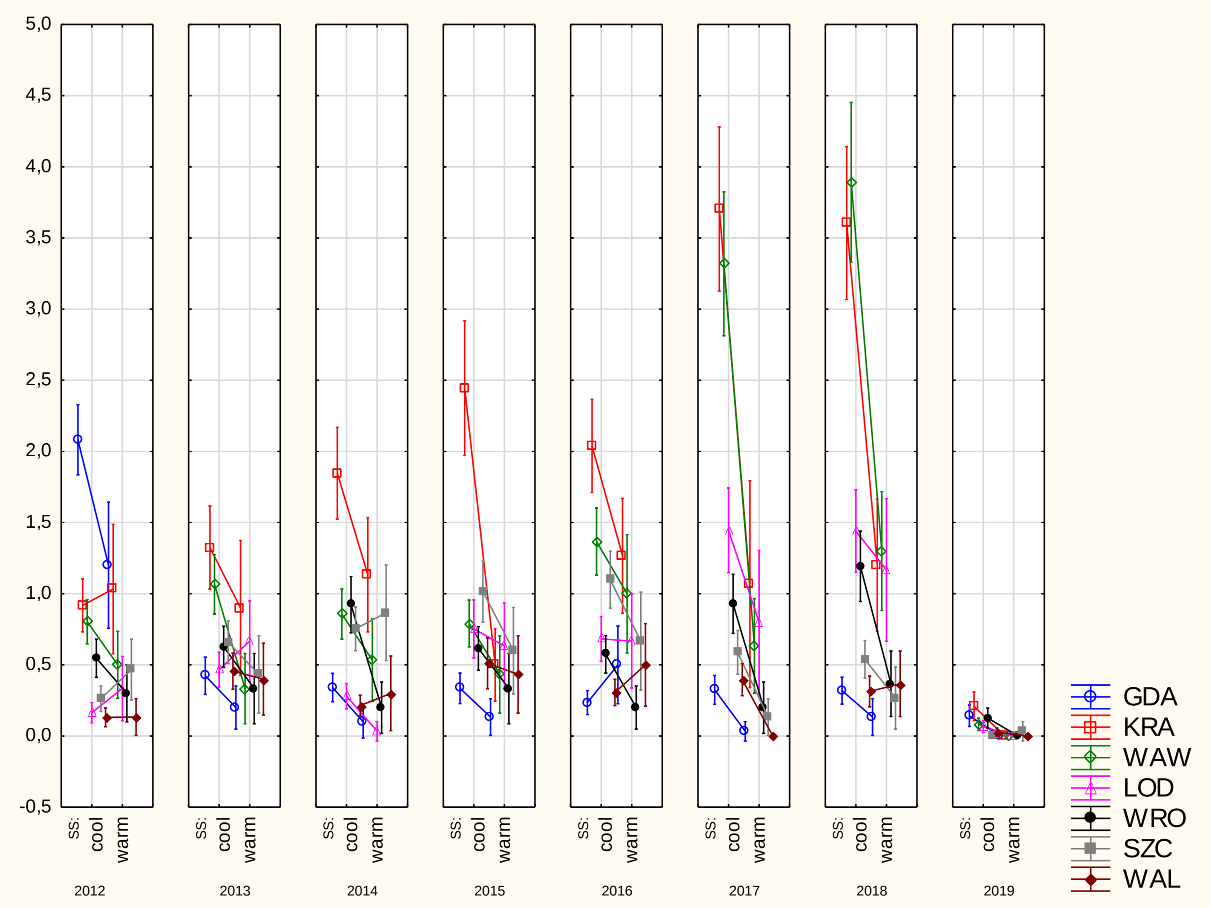

Table A) annual distribution of RSV cases

YEAR	GDA				KRA				WAW				LOD				WRO				SZC				WAL			
	Mean	Std.Err.	-0,95	0,95	Mean	Std.Err.	-0,95	0,95	Mean	Std.Err.	-0,95	0,95	Mean	Std.Err.	-0,95	0,95	Mean	Std.Err.	-0,95	0,95	Mean	Std.Err.	-0,95	0,95	Mean	Std.Err.	-0,95	0,95
2012	1,96	0,11	1,73	2,18	0,93	0,09	0,76	1,11	0,76	0,07	0,62	0,90	0,19	0,03	0,12	0,26	0,51	0,06	0,39	0,63	0,29	0,04	0,21	0,37	0,13	0,03	0,07	0,19
2013	0,39	0,06	0,28	0,51	1,26	0,13	1,01	1,52	0,96	0,09	0,78	1,15	0,50	0,06	0,38	0,61	0,58	0,07	0,46	0,71	0,63	0,07	0,49	0,76	0,45	0,06	0,33	0,56
2014	0,31	0,04	0,22	0,39	1,75	0,14	1,46	2,03	0,81	0,08	0,65	0,97	0,25	0,04	0,17	0,32	0,82	0,09	0,65	0,99	0,77	0,07	0,63	0,91	0,23	0,04	0,15	0,30
2015	0,31	0,05	0,21	0,40	2,17	0,21	1,75	2,59	0,74	0,07	0,59	0,89	0,74	0,09	0,56	0,92	0,58	0,07	0,44	0,71	0,96	0,10	0,77	1,15	0,50	0,08	0,34	0,66
2016	0,27	0,04	0,19	0,35	1,93	0,15	1,64	2,22	1,31	0,11	1,10	1,52	0,68	0,07	0,54	0,82	0,52	0,06	0,40	0,64	1,04	0,09	0,86	1,22	0,33	0,04	0,24	0,42
2017	0,28	0,05	0,19	0,37	3,33	0,26	2,81	3,85	2,94	0,23	2,48	3,39	1,35	0,13	1,09	1,62	0,83	0,09	0,64	1,01	0,52	0,07	0,39	0,66	0,34	0,05	0,24	0,44
2018	0,29	0,04	0,21	0,38	3,26	0,24	2,79	3,74	3,52	0,25	3,02	4,02	1,40	0,13	1,14	1,66	1,08	0,11	0,86	1,29	0,50	0,06	0,38	0,62	0,32	0,05	0,22	0,42
2019	0,12	0,03	0,06	0,19	0,18	0,04	0,09	0,27	0,07	0,02	0,03	0,11	0,06	0,02	0,02	0,09	0,11	0,03	0,05	0,17	0,00	0,00	0,00	0,01	0,02	0,01	0,00	0,05

Table B) seasonal distribution of RSV cases

SEASON	GDA				KRA				WAW				LOD				WRO				SZC				WAL			
	Mean	Std.Err.	-95,00%	95,00%	Mean	Std.Err.	-95,00%	95,00%	Mean	Std.Err.	-95,00%	95,00%	Mean	Std.Err.	-95,00%	95,00%	Mean	Std.Err.	-95,00%	95,00%	Mean	Std.Err.	-95,00%	95,00%	Mean	Std.Err.	-95,00%	95,00%
cool	0,53	0,03	0,47	0,58	2,01	0,08	1,86	2,16	1,52	0,06	1,40	1,65	0,66	0,04	0,59	0,73	0,69	0,03	0,63	0,75	0,61	0,03	0,56	0,67	0,29	0,02	0,26	0,33
warm	0,29	0,04	0,20	0,37	0,89	0,08	0,73	1,04	0,59	0,06	0,48	0,70	0,54	0,06	0,42	0,66	0,24	0,03	0,17	0,31	0,43	0,05	0,34	0,52	0,27	0,04	0,19	0,34

Table C) annual and seasonal distribution of RSV cases

YEAR	SEASON	GDA				KRA				WAW				LOD				WRO				SZC				WAL			
		Mean	Std.Err.	-0,95	0,95	Mean	Std.Err.	-0,95	0,95	Mean	Std.Err.	-0,95	0,95	Mean	Std.Err.	-0,95	0,95	Mean	Std.Err.	-0,95	0,95	Mean	Std.Err.	-0,95	0,95	Mean	Std.Err.	-0,95	0,95
2012	cool	2,08	0,12	1,84	2,33	0,92	0,09	0,73	1,10	0,80	0,08	0,65	0,96	0,16	0,04	0,09	0,23	0,55	0,07	0,41	0,68	0,26	0,05	0,17	0,35	0,13	0,03	0,07	0,20
2012	warm	1,20	0,22	0,76	1,64	1,03	0,22	0,58	1,49	0,50	0,11	0,26	0,74	0,33	0,11	0,11	0,56	0,30	0,10	0,10	0,50	0,47	0,10	0,25	0,68	0,13	0,06	0,00	0,26
2013	cool	0,42	0,07	0,29	0,55	1,32	0,15	1,03	1,62	1,07	0,11	0,86	1,27	0,47	0,06	0,35	0,59	0,63	0,07	0,48	0,77	0,66	0,08	0,51	0,81	0,46	0,06	0,33	0,58
2013	warm	0,20	0,07	0,05	0,35	0,90	0,23	0,43	1,37	0,33	0,12	0,09	0,58	0,67	0,14	0,38	0,95	0,33	0,12	0,09	0,58	0,43	0,13	0,16	0,71	0,40	0,12	0,15	0,65
2014	cool	0,34	0,05	0,24	0,44	1,85	0,16	1,52	2,17	0,86	0,09	0,68	1,03	0,28	0,05	0,19	0,37	0,92	0,10	0,73	1,12	0,75	0,08	0,60	0,91	0,21	0,04	0,14	0,29
2014	warm	0,10	0,06	-0,01	0,21	1,13	0,20	0,73	1,53	0,53	0,14	0,24	0,82	0,03	0,03	-0,03	0,10	0,20	0,09	0,02	0,38	0,87	0,16	0,53	1,20	0,30	0,13	0,04	0,56
2015	cool	0,34	0,05	0,23	0,44	2,45	0,24	1,97	2,92	0,79	0,08	0,63	0,96	0,75	0,10	0,55	0,96	0,62	0,08	0,46	0,77	1,02	0,11	0,80	1,23	0,51	0,09	0,33	0,69
2015	warm	0,13	0,06	0,00	0,26	0,50	0,12	0,25	0,75	0,43	0,13	0,16	0,71	0,63	0,15	0,33	0,94	0,33	0,12	0,09	0,58	0,60	0,15	0,30	0,90	0,43	0,13	0,16	0,71
2016	cool	0,23	0,04	0,15	0,32	2,04	0,17	1,71	2,37	1,37	0,12	1,13	1,60	0,68	0,08	0,53	0,84	0,57	0,07	0,44	0,71	1,10	0,10	0,90	1,30	0,31	0,05	0,21	0,40
2016	warm	0,50	0,13	0,23	0,77	1,27	0,20	0,86	1,67	1,00	0,20	0,58	1,42	0,67	0,16	0,34	1,00	0,20	0,07	0,05	0,35	0,67	0,17	0,32	1,01	0,50	0,14	0,21	0,79
2017	cool	0,32	0,05	0,22	0,43	3,70	0,29	3,13	4,28	3,32	0,26	2,81	3,82	1,45	0,15	1,15	1,74	0,93	0,11	0,72	1,14	0,59	0,08	0,43	0,74	0,40	0,06	0,28	0,51
2017	warm	0,03	0,03	-0,03	0,10	1,07	0,36	0,34	1,79	0,63	0,16	0,30	0,97	0,80	0,25	0,30	1,30	0,20	0,09	0,02	0,38	0,13	0,06	0,00	0,26	0,00			
2018	cool	0,32	0,05	0,22	0,41	3,60	0,27	3,07	4,14	3,89	0,28	3,33	4,45	1,44	0,15	1,15	1,73	1,19	0,13	0,95	1,44	0,54	0,07	0,41	0,67	0,31	0,05	0,20	0,42
2018	warm	0,13	0,06	0,00	0,26	1,20	0,23	0,74	1,66	1,30	0,20	0,88	1,72	1,17	0,24	0,67	1,67	0,37	0,11	0,14	0,60	0,27	0,11	0,05	0,48	0,37	0,11	0,14	0,60
2019	cool	0,14	0,04	0,07	0,22	0,21	0,05	0,11	0,31	0,08	0,02	0,04	0,13	0,07	0,02	0,02	0,11	0,13	0,04	0,06	0,20	0,00				0,03	0,01	0,00	0,06
2019	warm	0,00				0,00				0,00				0,00				0,00				0,03	0,03	-0,03	0,10	0,00			
