# Supplementary material for: Air Pollutants’ Concentrations Are Associated with Increased Number of RSV Hospitalizations in Polish Children
Source: J Clin Med. 2021 Jul 22;10(15):3224. doi: 10.3390/jcm10153224 (PMC8348891; doi:10.3390/jcm10153224)
Supplement: Supplementary file 1 [file jcm-10-03224-s001.zip › Supplementary materials 2 revised.pdf]

Supplementary materials 2. The annual boxplots of air pollutants' ($PM_{2.5}$, PM_{10}, NO_2) concentrations in the studied cities throughout the analysed period (2012-2019). Abbreviations for the cities: GDA- Gdansk, WAW- Warsaw, KRA-Krakow, WRO- Wroclaw, LOD- Lodz, SZC- Szczecin, WAL- Walbrzych

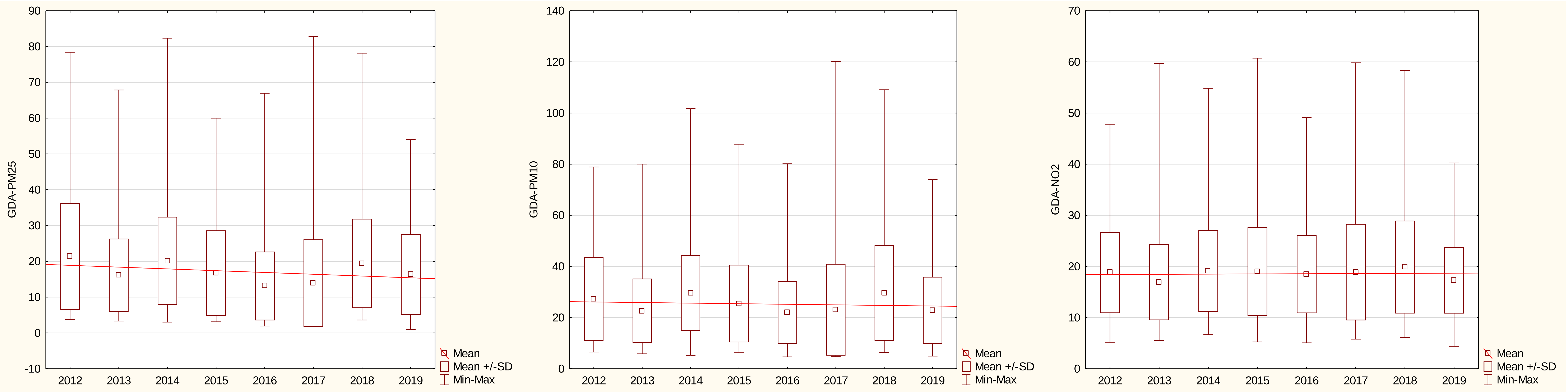

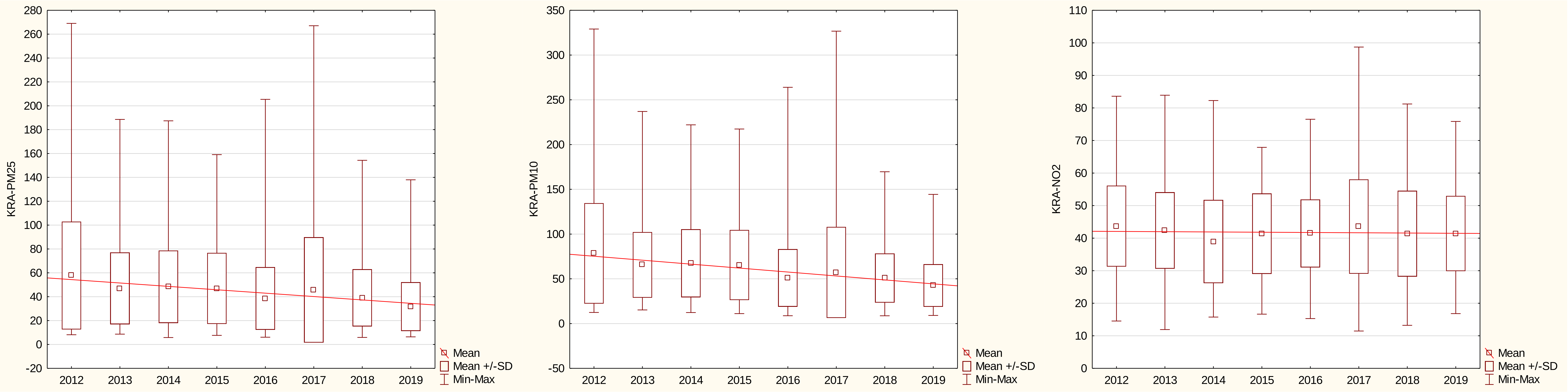

KRA-PM25
KRA-PM10
KRA-NO2
2012
2013
2014
2015
2016
2017
2018
2019
Mean
Mean +/-SD
Min-Max

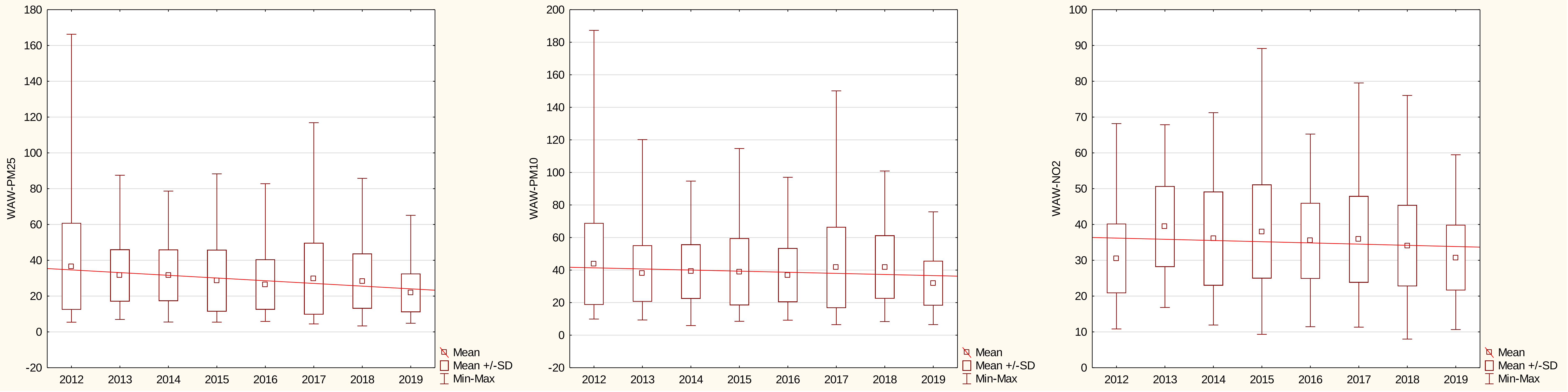
WAW-PM25
180
160
140
120
100
80
60
40
20
0
-20
2012
2013
2014
2015
2016
2017
2018
2019
Mean
Mean +/-SD
Min-Max
WAW-PM10
200
180
160
140
120
100
80
60
40
20
0
-20
2012
2013
2014
2015
2016
2017
2018
2019
Mean
Mean +/-SD
Min-Max
WAW-NO2
100
90
80
70
60
50
40
30
20
10
0
2012
2013
2014
2015
2016
2017
2018
2019
Mean
Mean +/-SD
Min-Max

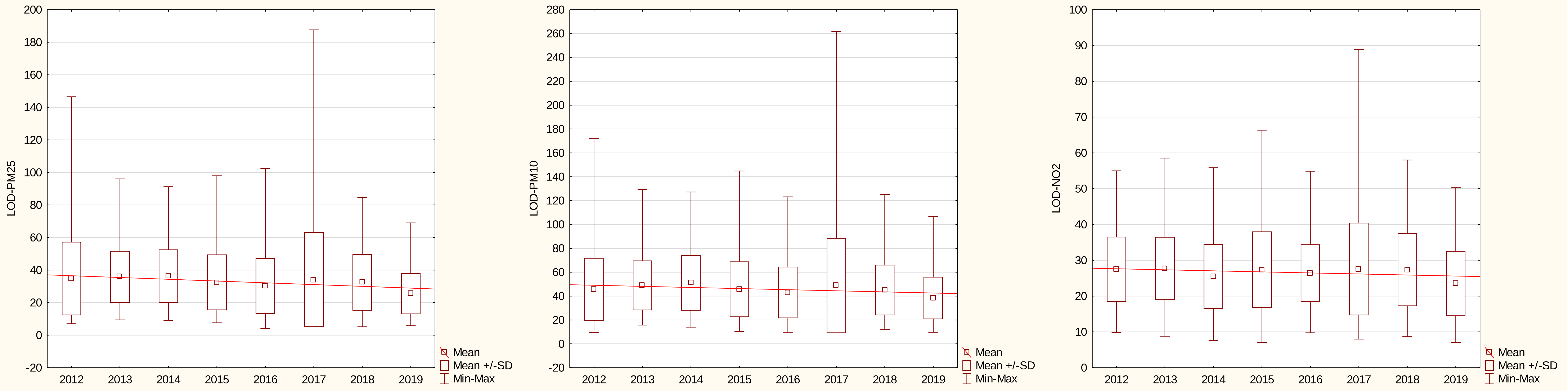
LOD-PM25
LOD-PM10
LOD-NO2
2012
2013
2014
2015
2016
2017
2018
2019
Mean
Mean +/-SD
Min-Max

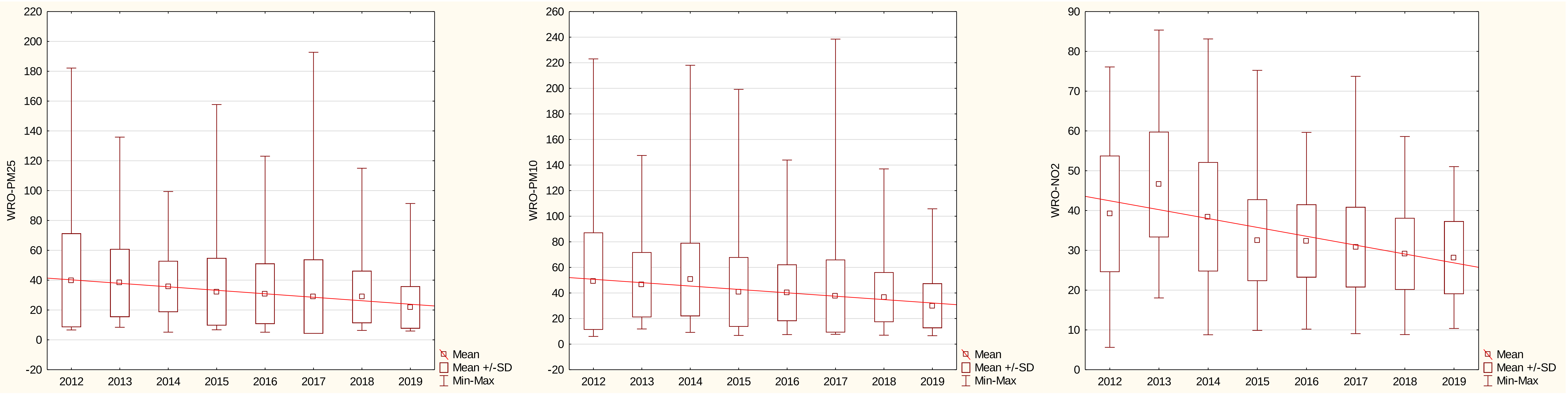

WRO-PM25
220
200
180
160
140
120
100
80
60
40
20
0
-20
2012
2013
2014
2015
2016
2017
2018
2019
Mean
Mean +/-SD
Min-Max
WRO-PM10
260
240
220
200
180
160
140
120
100
80
60
40
20
0
-20
2012
2013
2014
2015
2016
2017
2018
2019
Mean
Mean +/-SD
Min-Max
WRO-NO2
90
80
70
60
50
40
30
20
10
0
2012
2013
2014
2015
2016
2017
2018
2019
Mean
Mean +/-SD
Min-Max

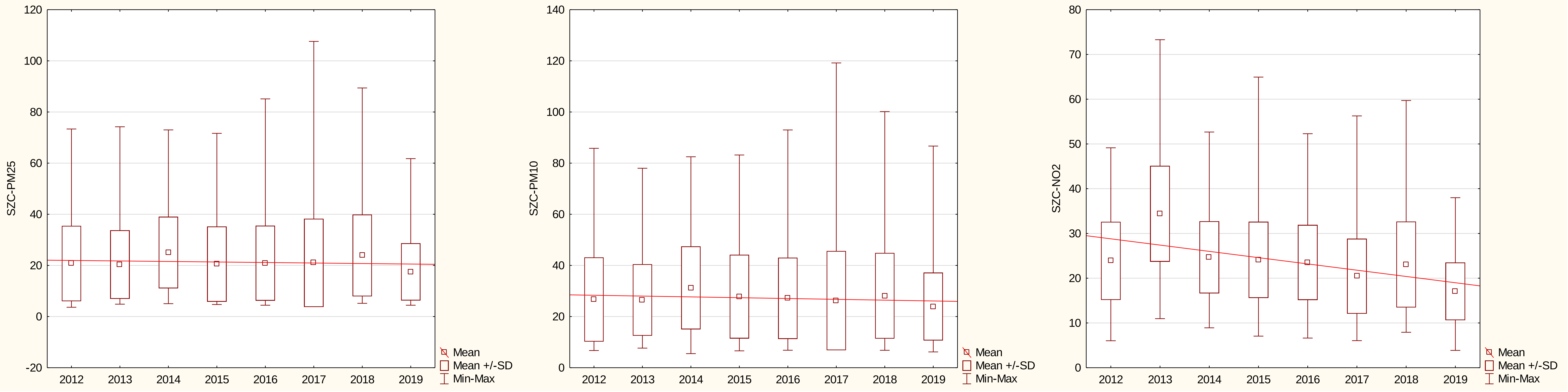

SZC-PM25
SZC-PM10
SZC-NO2
2012
2013
2014
2015
2016
2017
2018
2019
Mean
Mean +/-SD
Min-Max

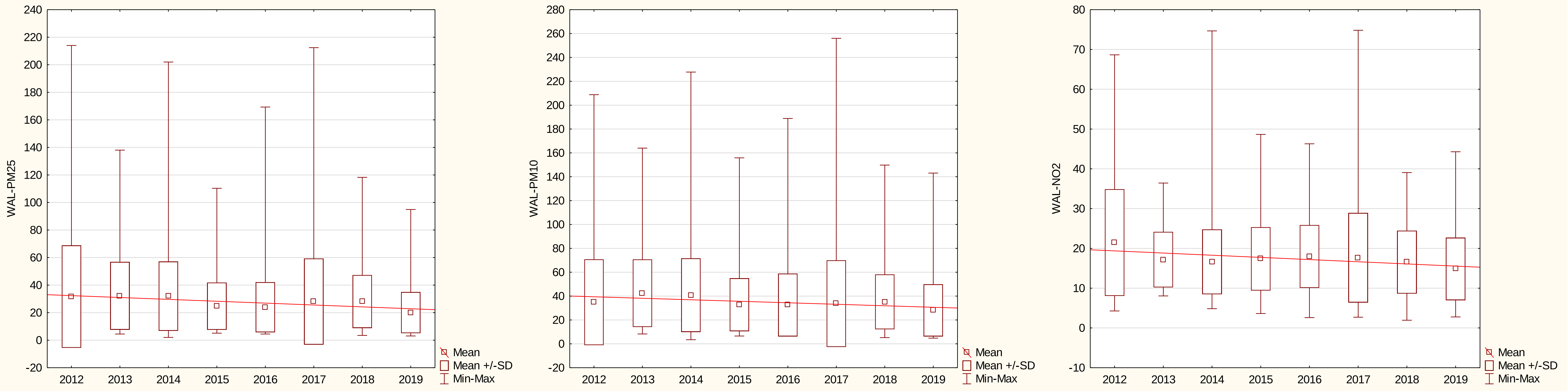

WAL-PM25
240
220
200
180
160
140
120
100
80
60
40
20
0
-20
2012
2013
2014
2015
2016
2017
2018
2019
Mean
Mean +/-SD
Min-Max
WAL-PM10
280
260
240
220
200
180
160
140
120
100
80
60
40
20
0
-20
2012
2013
2014
2015
2016
2017
2018
2019
Mean
Mean +/-SD
Min-Max
WAL-NO2
80
70
60
50
40
30
20
10
0
-10
2012
2013
2014
2015
2016
2017
2018
2019
Mean
Mean +/-SD
Min-Max
