# Supplementary material for: Air Pollutants’ Concentrations Are Associated with Increased Number of RSV Hospitalizations in Polish Children
Source: J Clin Med. 2021 Jul 22;10(15):3224. doi: 10.3390/jcm10153224 (PMC8348891; doi:10.3390/jcm10153224)
Supplement: Supplementary file 1 [file jcm-10-03224-s001.zip › Supplementary materials 3.pdf]

Supplementary materials 3.

The exemplary results of the city to city comparisons of autocorrelation function (ACF) and partial autocorrelation function (PACF) for Warsaw (a- ACF for NO2, b- PACF for NO2, c- ACF for PM2.5, d- PACF for PM2.5, e- ACF for PM10, f- PACF for PM10) and Gdansk (g- ACF for NO2, h- PACF for NO2, i- ACF for PM2.5, j- PACF for PM2.5, k- ACF for PM10, l- PACF for PM10).

a)

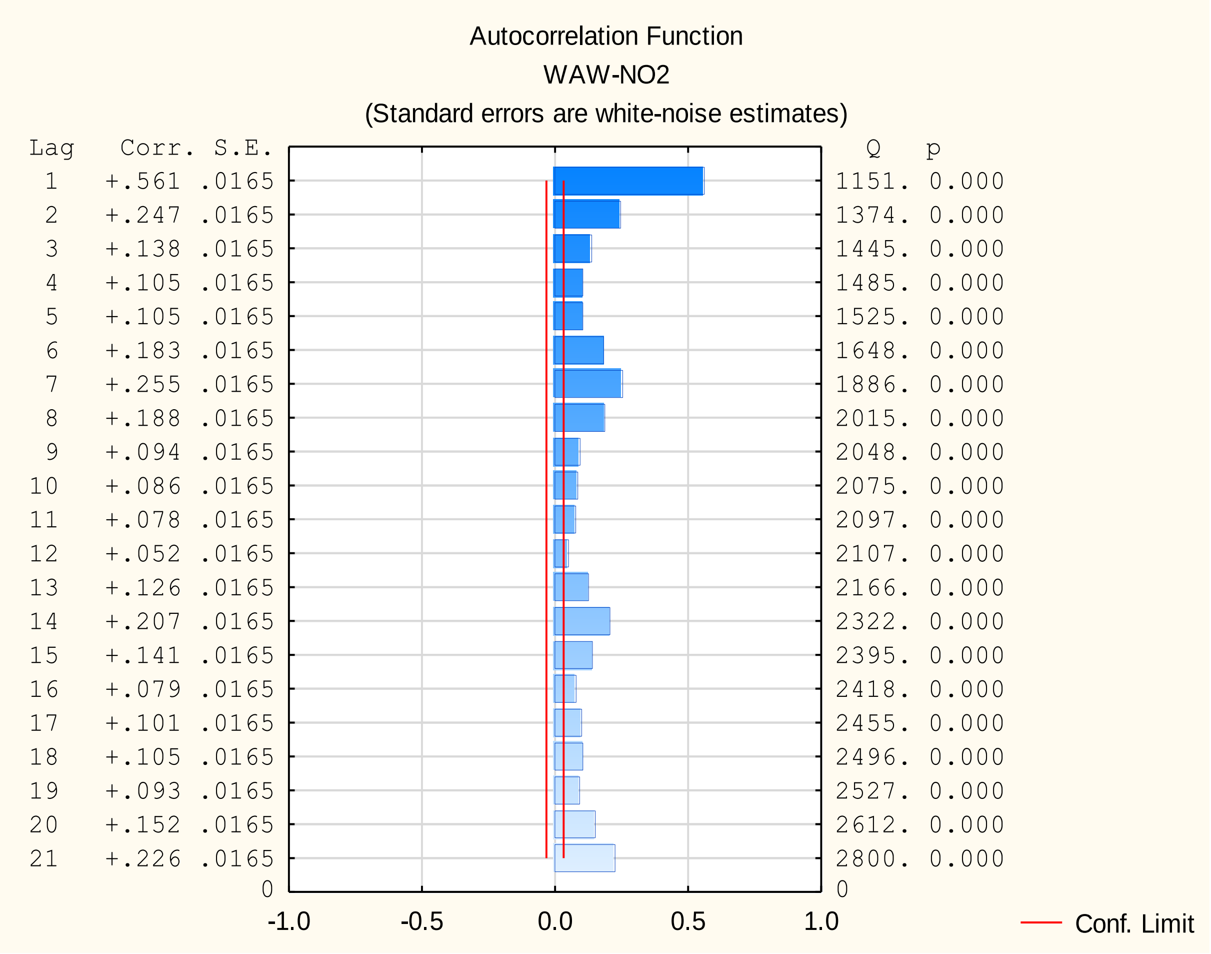

b)

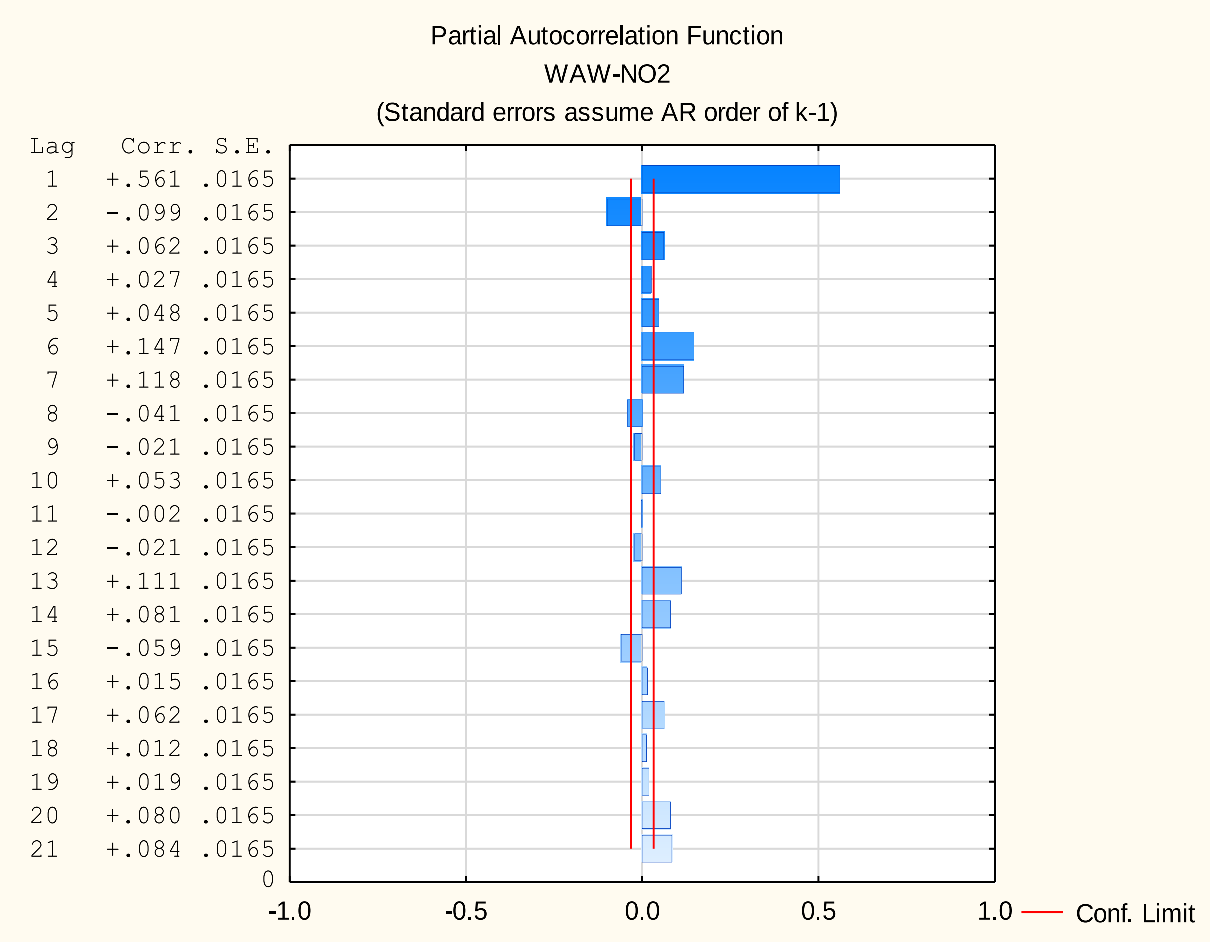

Partial Autocorrelation Function
WAW-NO2
(Standard errors assume AR order of k-1)
Lag Corr. S.E.
1 +.561 .0165
2 -.099 .0165
3 +.062 .0165
4 +.027 .0165
5 +.048 .0165
6 +.147 .0165
7 +.118 .0165
8 -.041 .0165
9 -.021 .0165
10 +.053 .0165
11 -.002 .0165
12 -.021 .0165
13 +.111 .0165
14 +.081 .0165
15 -.059 .0165
16 +.015 .0165
17 +.062 .0165
18 +.012 .0165
19 +.019 .0165
20 +.080 .0165
21 +.084 .0165
0
-1.0
-0.5
0.0
0.5
1.0
Conf. Limit

c)

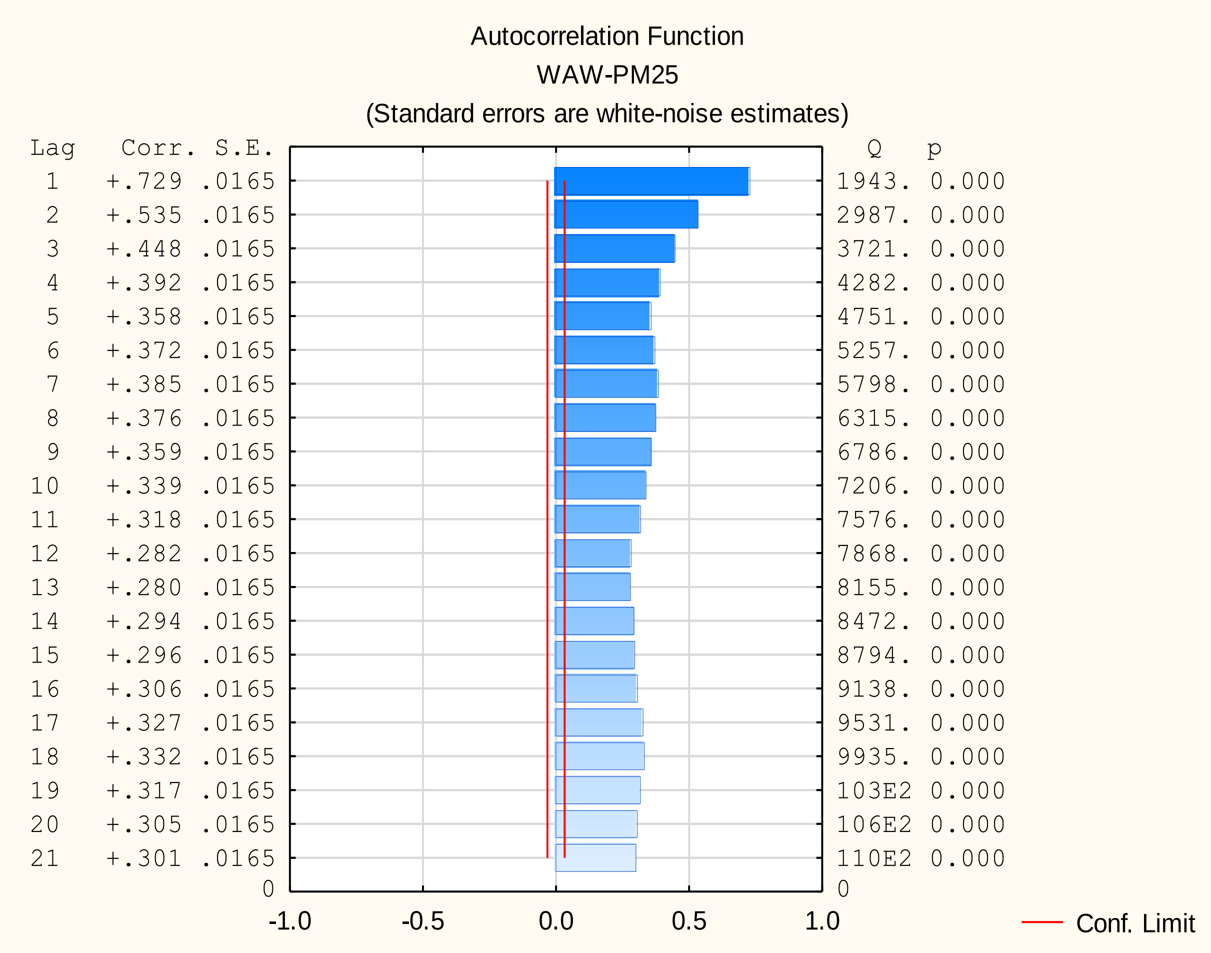
Autocorrelation Function
WAW-PM25
(Standard errors are white-noise estimates)
Lag Corr. S.E.
1 +.729 .0165
2 +.535 .0165
3 +.448 .0165
4 +.392 .0165
5 +.358 .0165
6 +.372 .0165
7 +.385 .0165
8 +.376 .0165
9 +.359 .0165
10 +.339 .0165
11 +.318 .0165
12 +.282 .0165
13 +.280 .0165
14 +.294 .0165
15 +.296 .0165
16 +.306 .0165
17 +.327 .0165
18 +.332 .0165
19 +.317 .0165
20 +.305 .0165
21 +.301 .0165
0
Q p
1943. 0.000
2987. 0.000
3721. 0.000
4282. 0.000
4751. 0.000
5257. 0.000
5798. 0.000
6315. 0.000
6786. 0.000
7206. 0.000
7576. 0.000
7868. 0.000
8155. 0.000
8472. 0.000
8794. 0.000
9138. 0.000
9531. 0.000
9935. 0.000
103E2 0.000
106E2 0.000
110E2 0.000
0
-1.0
-0.5
0.0
0.5
1.0
Conf. Limit

d)

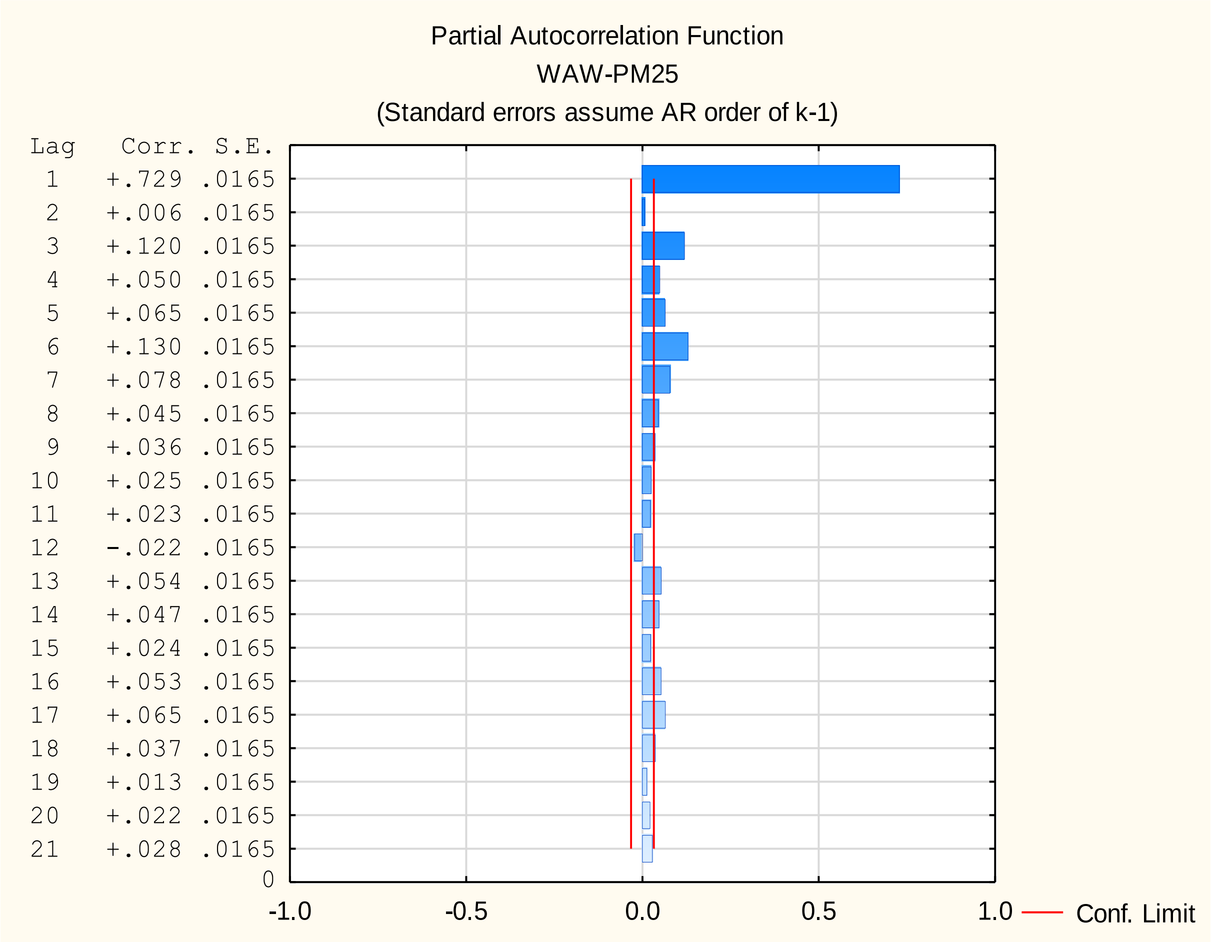
Partial Autocorrelation Function
WAW-PM25
(Standard errors assume AR order of k-1)
Lag Corr. S.E.
1 +.729 .0165
2 +.006 .0165
3 +.120 .0165
4 +.050 .0165
5 +.065 .0165
6 +.130 .0165
7 +.078 .0165
8 +.045 .0165
9 +.036 .0165
10 +.025 .0165
11 +.023 .0165
12 -.022 .0165
13 +.054 .0165
14 +.047 .0165
15 +.024 .0165
16 +.053 .0165
17 +.065 .0165
18 +.037 .0165
19 +.013 .0165
20 +.022 .0165
21 +.028 .0165
0
-1.0
-0.5
0.0
0.5
1.0
Conf. Limit

e)

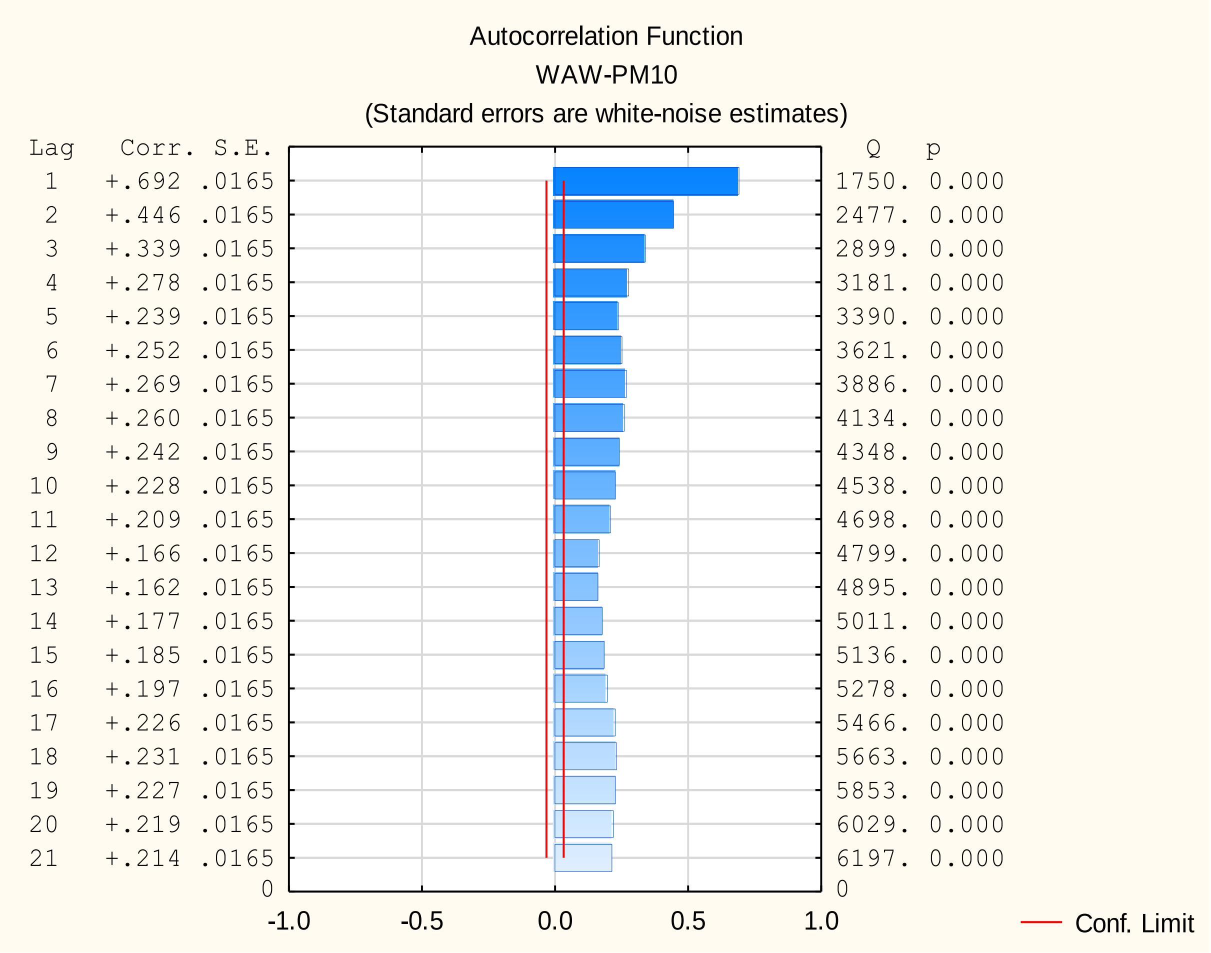

Autocorrelation Function
WAW-PM10
(Standard errors are white-noise estimates)
Lag Corr. S.E.
Q p
1 +.692 .0165 1750. 0.000
2 +.446 .0165 2477. 0.000
3 +.339 .0165 2899. 0.000
4 +.278 .0165 3181. 0.000
5 +.239 .0165 3390. 0.000
6 +.252 .0165 3621. 0.000
7 +.269 .0165 3886. 0.000
8 +.260 .0165 4134. 0.000
9 +.242 .0165 4348. 0.000
10 +.228 .0165 4538. 0.000
11 +.209 .0165 4698. 0.000
12 +.166 .0165 4799. 0.000
13 +.162 .0165 4895. 0.000
14 +.177 .0165 5011. 0.000
15 +.185 .0165 5136. 0.000
16 +.197 .0165 5278. 0.000
17 +.226 .0165 5466. 0.000
18 +.231 .0165 5663. 0.000
19 +.227 .0165 5853. 0.000
20 +.219 .0165 6029. 0.000
21 +.214 .0165 6197. 0.000
0
0
-1.0
-0.5
0.0
0.5
1.0
Conf. Limit

f)

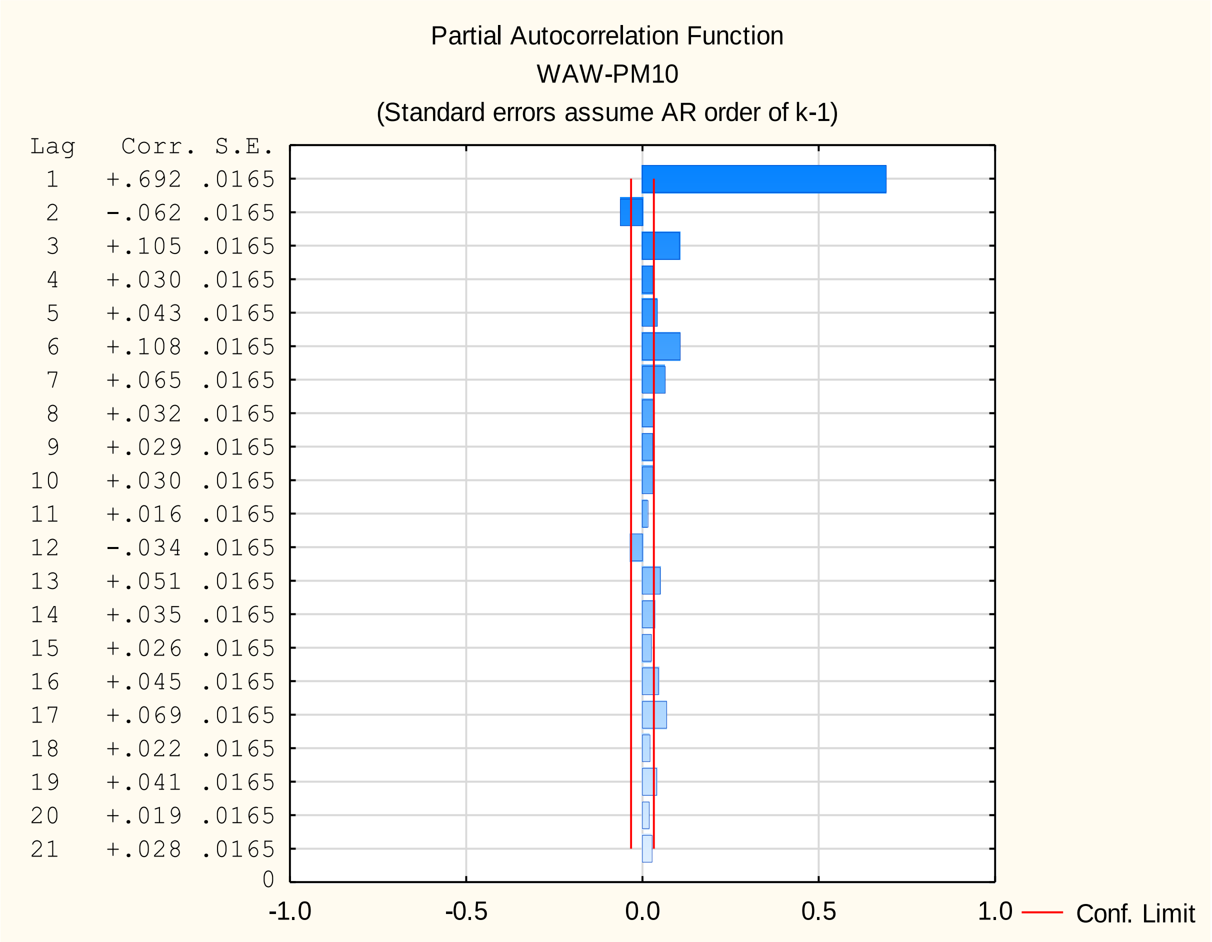
Partial Autocorrelation Function
WAW-PM10
(Standard errors assume AR order of k-1)
Lag Corr. S.E.
1 +.692 .0165
2 -.062 .0165
3 +.105 .0165
4 +.030 .0165
5 +.043 .0165
6 +.108 .0165
7 +.065 .0165
8 +.032 .0165
9 +.029 .0165
10 +.030 .0165
11 +.016 .0165
12 -.034 .0165
13 +.051 .0165
14 +.035 .0165
15 +.026 .0165
16 +.045 .0165
17 +.069 .0165
18 +.022 .0165
19 +.041 .0165
20 +.019 .0165
21 +.028 .0165
0
-1.0
-0.5
0.0
0.5
1.0
Conf. Limit

g)

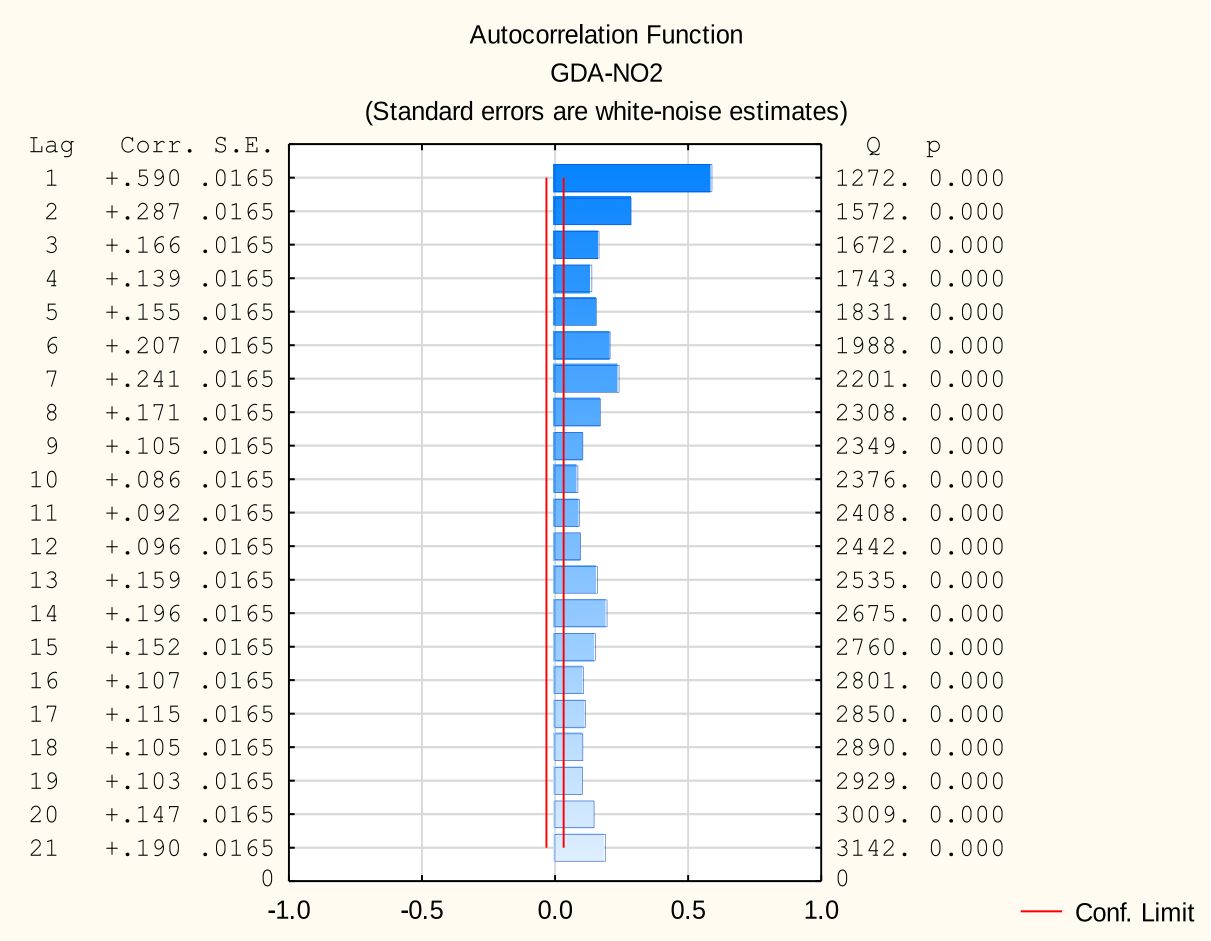
Autocorrelation Function
GDA-NO2
(Standard errors are white-noise estimates)
Lag Corr. S.E. Q p
1 +.590 .0165 1272. 0.000
2 +.287 .0165 1572. 0.000
3 +.166 .0165 1672. 0.000
4 +.139 .0165 1743. 0.000
5 +.155 .0165 1831. 0.000
6 +.207 .0165 1988. 0.000
7 +.241 .0165 2201. 0.000
8 +.171 .0165 2308. 0.000
9 +.105 .0165 2349. 0.000
10 +.086 .0165 2376. 0.000
11 +.092 .0165 2408. 0.000
12 +.096 .0165 2442. 0.000
13 +.159 .0165 2535. 0.000
14 +.196 .0165 2675. 0.000
15 +.152 .0165 2760. 0.000
16 +.107 .0165 2801. 0.000
17 +.115 .0165 2850. 0.000
18 +.105 .0165 2890. 0.000
19 +.103 .0165 2929. 0.000
20 +.147 .0165 3009. 0.000
21 +.190 .0165 3142. 0.000
0
0
-1.0
-0.5
0.0
0.5
1.0
Conf. Limit

h)

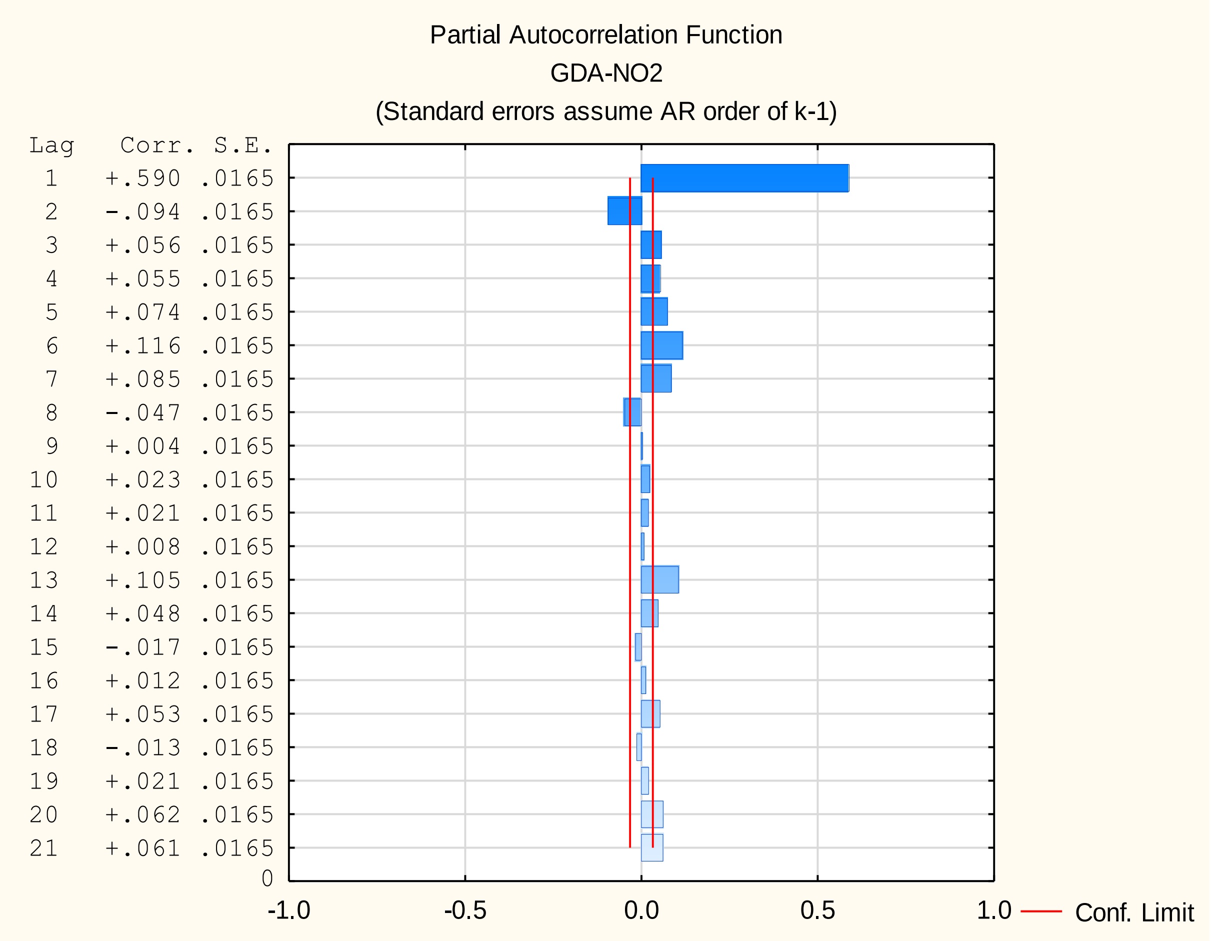

Partial Autocorrelation Function
GDA-NO2
(Standard errors assume AR order of k-1)
Lag Corr. S.E.
1 +.590 .0165
2 -.094 .0165
3 +.056 .0165
4 +.055 .0165
5 +.074 .0165
6 +.116 .0165
7 +.085 .0165
8 -.047 .0165
9 +.004 .0165
10 +.023 .0165
11 +.021 .0165
12 +.008 .0165
13 +.105 .0165
14 +.048 .0165
15 -.017 .0165
16 +.012 .0165
17 +.053 .0165
18 -.013 .0165
19 +.021 .0165
20 +.062 .0165
21 +.061 .0165
0
-1.0
-0.5
0.0
0.5
1.0
Conf. Limit

i)

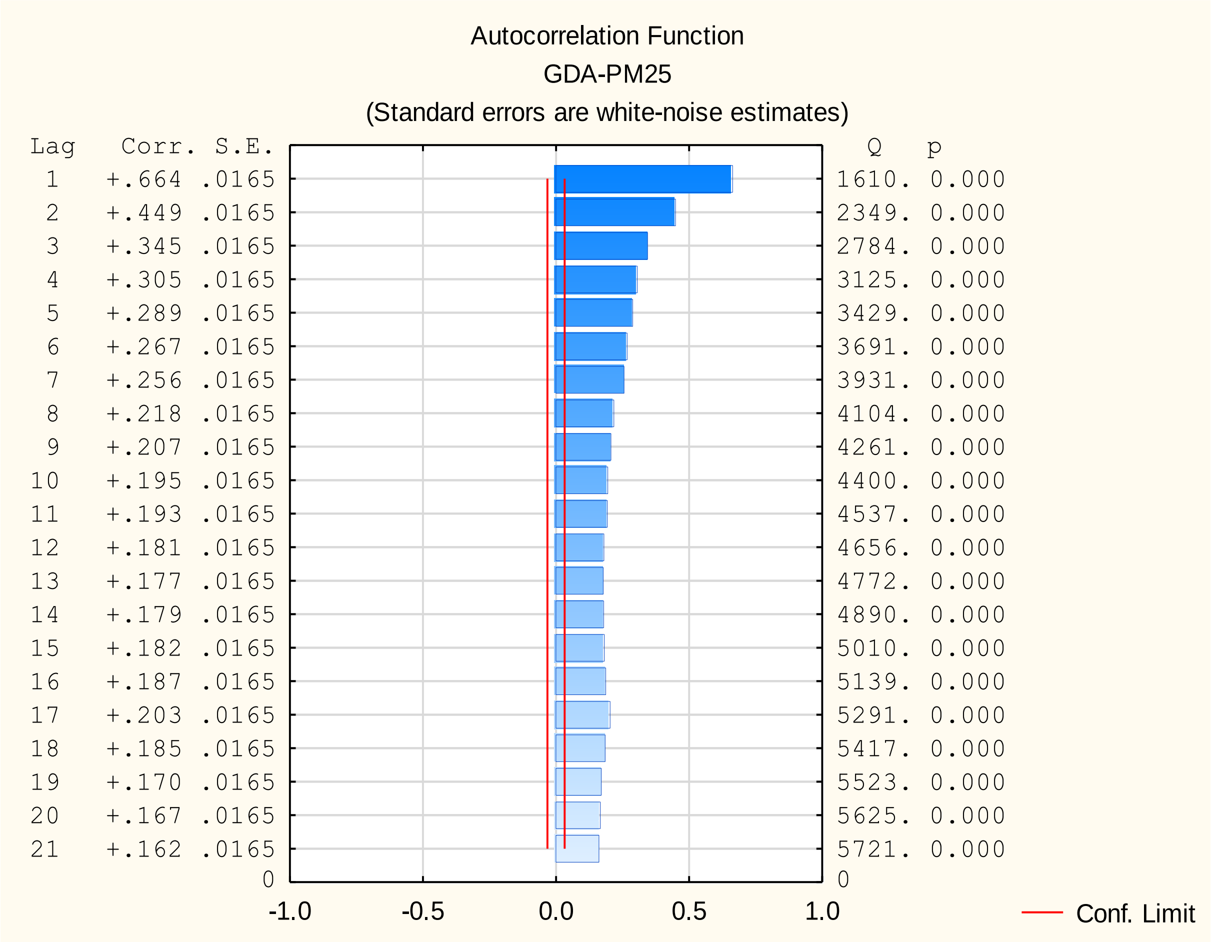
Autocorrelation Function
GDA-PM25
(Standard errors are white-noise estimates)
Lag Corr. S.E.
1 +.664 .0165
2 +.449 .0165
3 +.345 .0165
4 +.305 .0165
5 +.289 .0165
6 +.267 .0165
7 +.256 .0165
8 +.218 .0165
9 +.207 .0165
10 +.195 .0165
11 +.193 .0165
12 +.181 .0165
13 +.177 .0165
14 +.179 .0165
15 +.182 .0165
16 +.187 .0165
17 +.203 .0165
18 +.185 .0165
19 +.170 .0165
20 +.167 .0165
21 +.162 .0165
0
Q p
1610. 0.000
2349. 0.000
2784. 0.000
3125. 0.000
3429. 0.000
3691. 0.000
3931. 0.000
4104. 0.000
4261. 0.000
4400. 0.000
4537. 0.000
4656. 0.000
4772. 0.000
4890. 0.000
5010. 0.000
5139. 0.000
5291. 0.000
5417. 0.000
5523. 0.000
5625. 0.000
5721. 0.000
0
-1.0
-0.5
0.0
0.5
1.0
Conf. Limit

j)

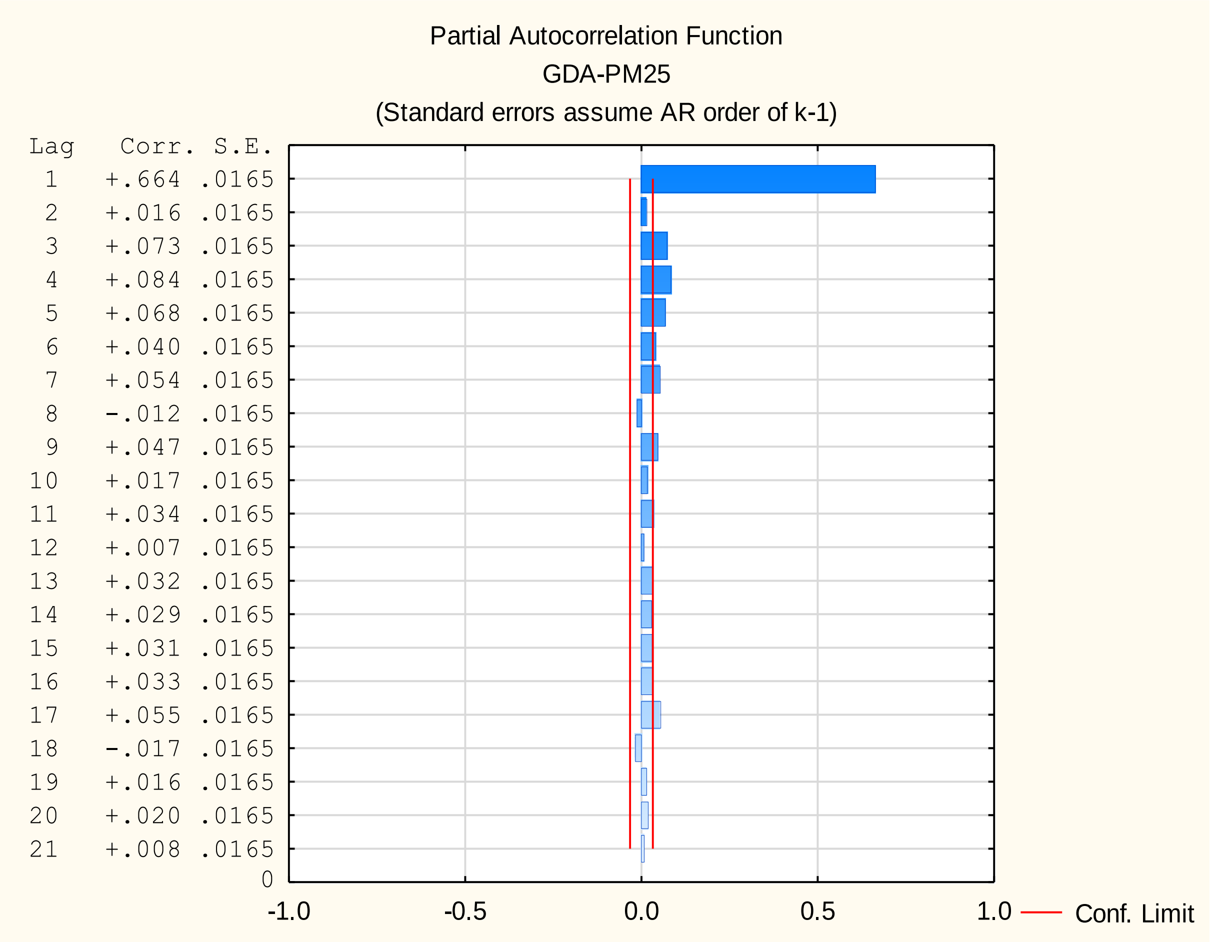
Partial Autocorrelation Function
GDA-PM25
(Standard errors assume AR order of k-1)
Lag Corr. S.E.
1 +.664 .0165
2 +.016 .0165
3 +.073 .0165
4 +.084 .0165
5 +.068 .0165
6 +.040 .0165
7 +.054 .0165
8 -.012 .0165
9 +.047 .0165
10 +.017 .0165
11 +.034 .0165
12 +.007 .0165
13 +.032 .0165
14 +.029 .0165
15 +.031 .0165
16 +.033 .0165
17 +.055 .0165
18 -.017 .0165
19 +.016 .0165
20 +.020 .0165
21 +.008 .0165
0
-1.0
-0.5
0.0
0.5
1.0
Conf. Limit

k)

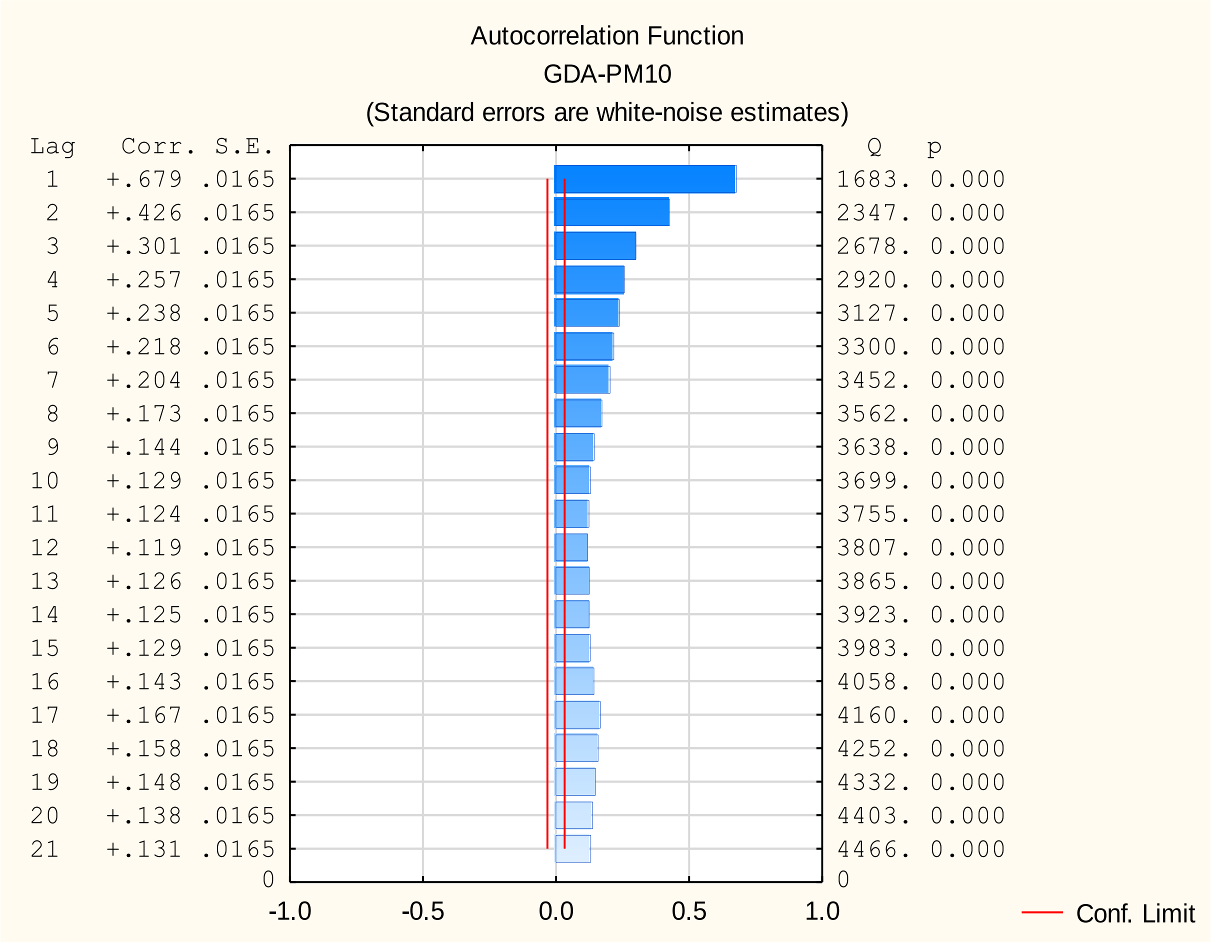
Autocorrelation Function
GDA-PM10
(Standard errors are white-noise estimates)
Lag Corr. S.E.
1 +.679 .0165
2 +.426 .0165
3 +.301 .0165
4 +.257 .0165
5 +.238 .0165
6 +.218 .0165
7 +.204 .0165
8 +.173 .0165
9 +.144 .0165
10 +.129 .0165
11 +.124 .0165
12 +.119 .0165
13 +.126 .0165
14 +.125 .0165
15 +.129 .0165
16 +.143 .0165
17 +.167 .0165
18 +.158 .0165
19 +.148 .0165
20 +.138 .0165
21 +.131 .0165
Q p
1683. 0.000
2347. 0.000
2678. 0.000
2920. 0.000
3127. 0.000
3300. 0.000
3452. 0.000
3562. 0.000
3638. 0.000
3699. 0.000
3755. 0.000
3807. 0.000
3865. 0.000
3923. 0.000
3983. 0.000
4058. 0.000
4160. 0.000
4252. 0.000
4332. 0.000
4403. 0.000
4466. 0.000
0
-1.0
-0.5
0.0
0.5
1.0
Conf. Limit

l)

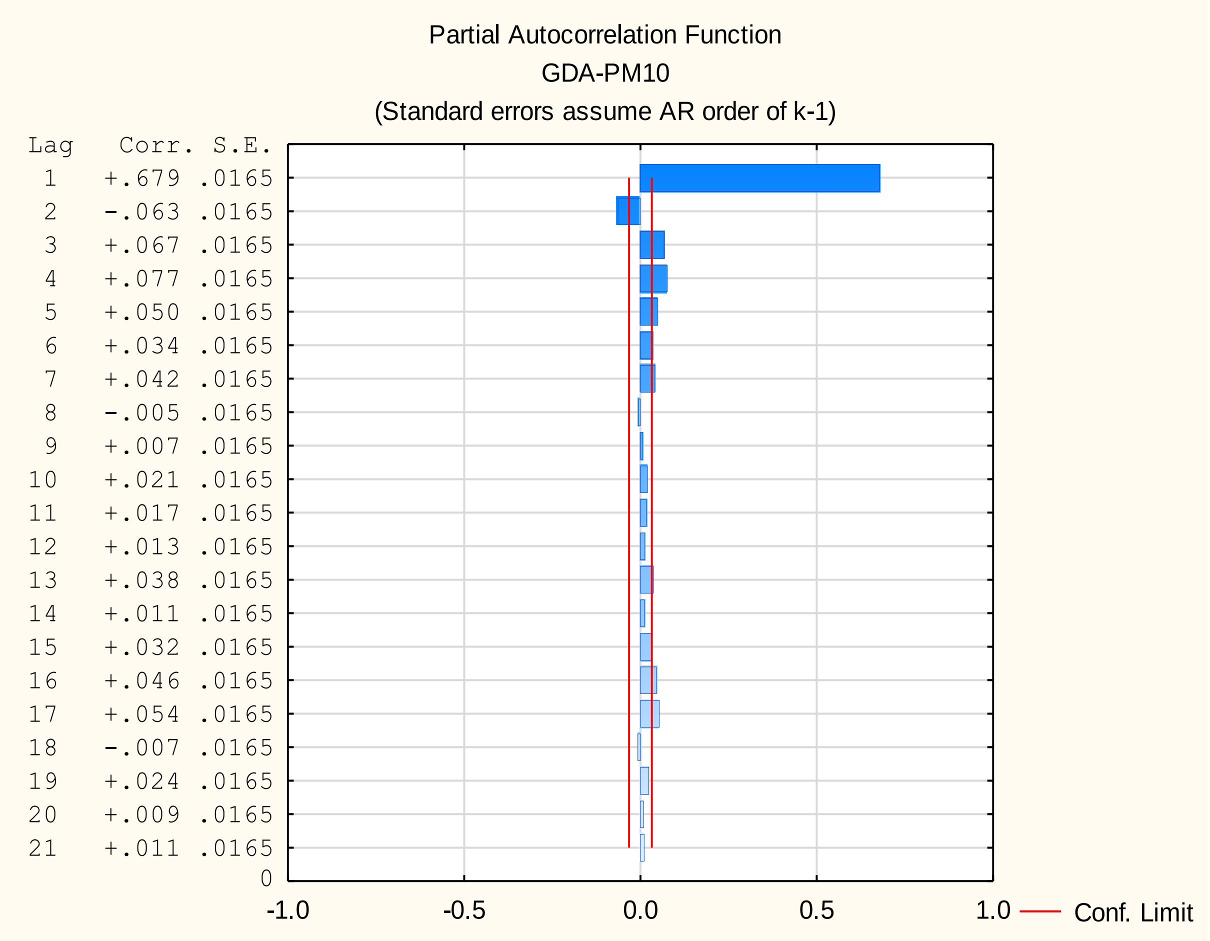
Partial Autocorrelation Function
GDA-PM10
(Standard errors assume AR order of k-1)
Lag Corr. S.E.
1 +.679 .0165
2 -.063 .0165
3 +.067 .0165
4 +.077 .0165
5 +.050 .0165
6 +.034 .0165
7 +.042 .0165
8 -.005 .0165
9 +.007 .0165
10 +.021 .0165
11 +.017 .0165
12 +.013 .0165
13 +.038 .0165
14 +.011 .0165
15 +.032 .0165
16 +.046 .0165
17 +.054 .0165
18 -.007 .0165
19 +.024 .0165
20 +.009 .0165
21 +.011 .0165
0
-1.0
-0.5
0.0
0.5
1.0
Conf. Limit
