# Supplementary material for: Air Pollutants’ Concentrations Are Associated with Increased Number of RSV Hospitalizations in Polish Children
Source: J Clin Med. 2021 Jul 22;10(15):3224. doi: 10.3390/jcm10153224 (PMC8348891; doi:10.3390/jcm10153224)
Supplement: Supplementary file 1 [file jcm-10-03224-s001.zip › Supplementary materials 4 revised.pdf]

Supplementary materials 4. A correlation between the air pollutants' concentrations (statistically significant coefficients are marked in red). Abbreviations for the cities: GDA- Gdansk, WAW- Warsaw, KRA-Krakow, WRO- Wroclaw, LOD- Lodz, SZC- Szczecin, WAL- Walbrzych

Variable	RSV_GDA	KRA RSV	WAW RSV	LOD RSV	WRO RSV	SZC RSV	WAL RSV
GDA-NO2	0,03						
GDA-PM25	0,11						
GDA-PM10	0,05						
KRA-NO2		0,13					
KRA-PM25		0,16					
KRA-PM10		0,13					
WAW-NO2			0,06				
WAW-PM25			0,12				
WAW-PM10			0,13				
LOD-NO2				0,14			
LOD-PM25				0,13			
LOD-PM10				0,12			
WRO-NO2					0,12		
WRO-PM25					0,17		
WRO-PM10					0,14		
SZC-NO2						0,15	
SZC-PM25						0,10	
SZC-PM10						0,09	
WAL-NO2							0,12
WAL-PM25							0,11
WAL-PM10							0,11
