# Supplementary material for: Air Pollutants’ Concentrations Are Associated with Increased Number of RSV Hospitalizations in Polish Children
Source: J Clin Med. 2021 Jul 22;10(15):3224. doi: 10.3390/jcm10153224 (PMC8348891; doi:10.3390/jcm10153224)
Supplement: Supplementary file 1 [file jcm-10-03224-s001.zip › Supplementary materials 5 revised.pdf]

Supplementary materials 5. The Pareto charts of the effects power (based on the F statistics) and corresponding scatterplots of the air pollutants concentrations and the RSV hospitalizations (sequentially: $PM_{2.5}$, PM_{10}, NO_2) during the RSV high-risk seasons (2012-2019) in selected study sites (the statistical significance is shown on the Pareto chart, air pollutant that were statistically insignificant are not shown in the Pareto chart, but a scatterplot is shown); a) Gdansk, b) Warsaw, c) Krakow, d) Lodz, e) Wroclaw, f) Szczecin, g) Walbrzych

A) Gdansk

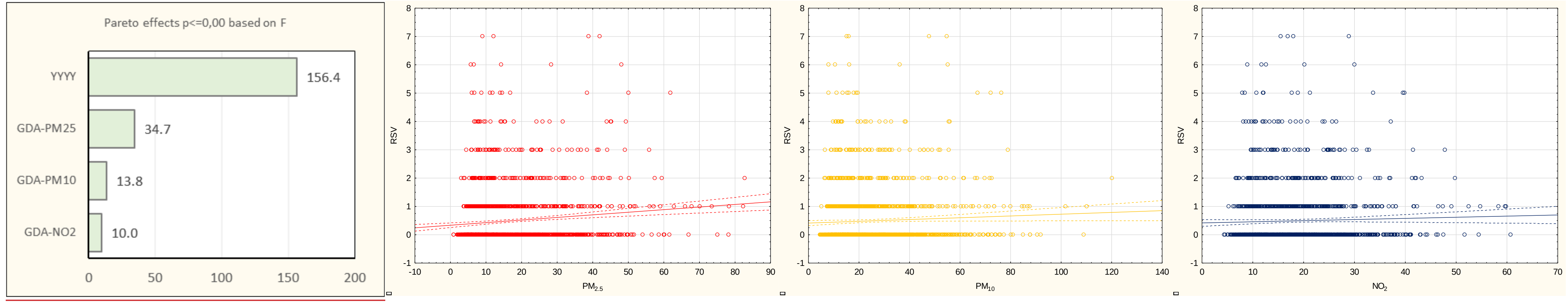

B) Warsaw

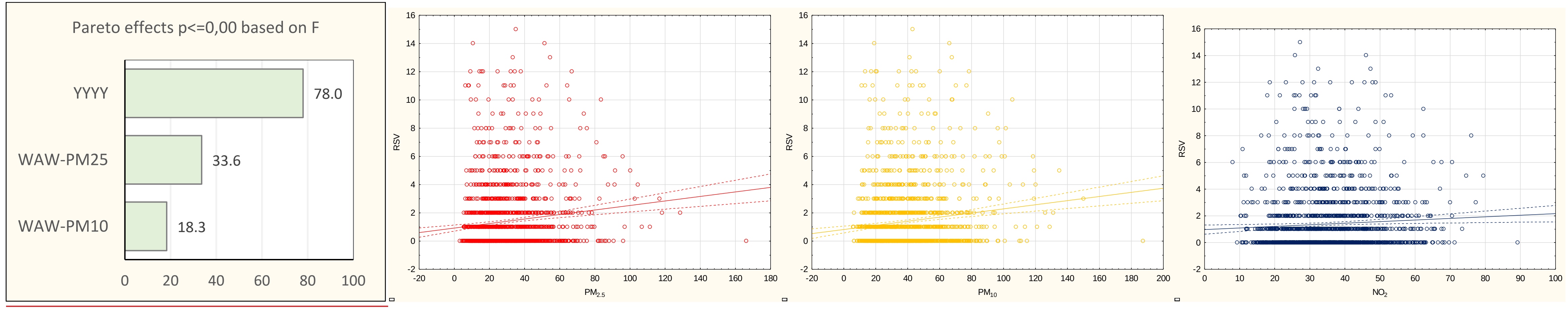

C) Krakow

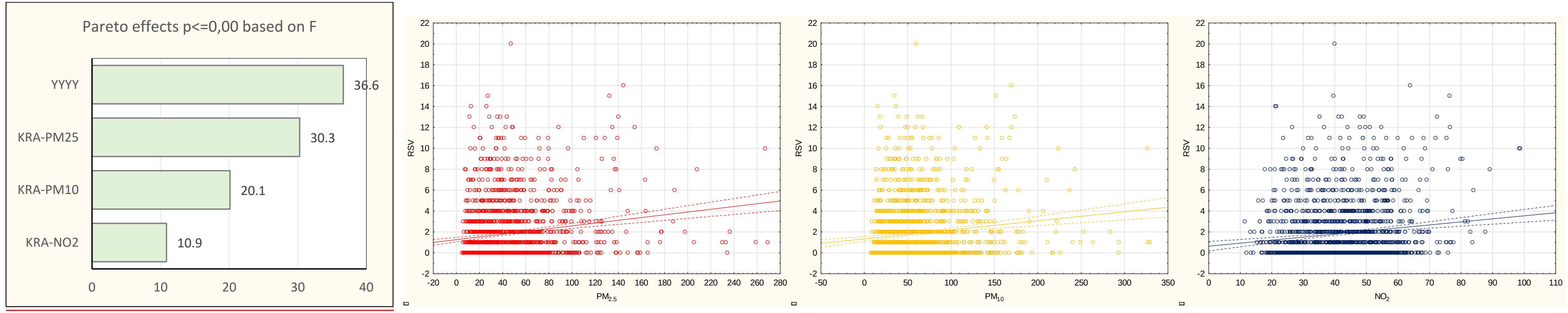

D) Lodz

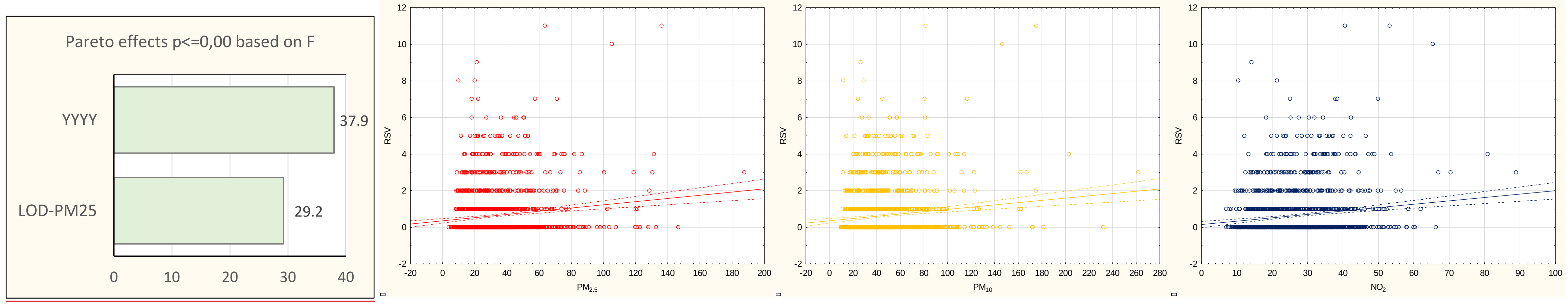

E) Wroclaw

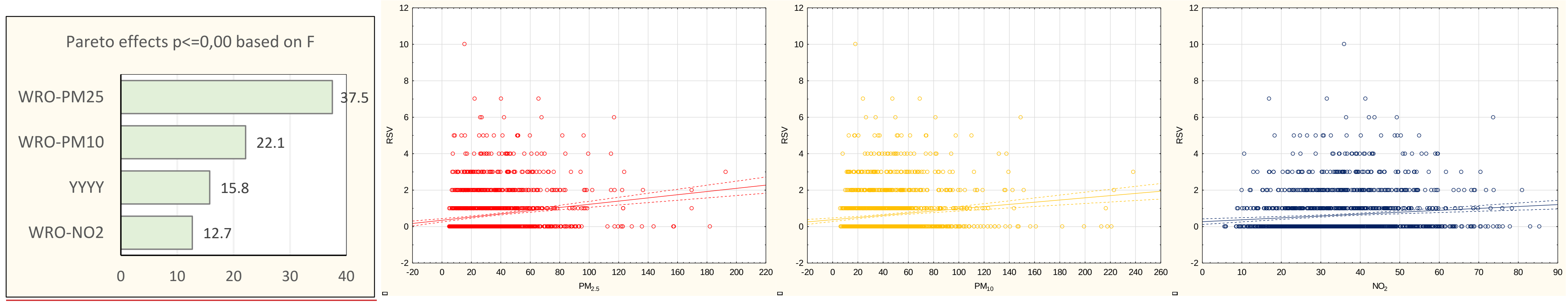

F) Szczecin

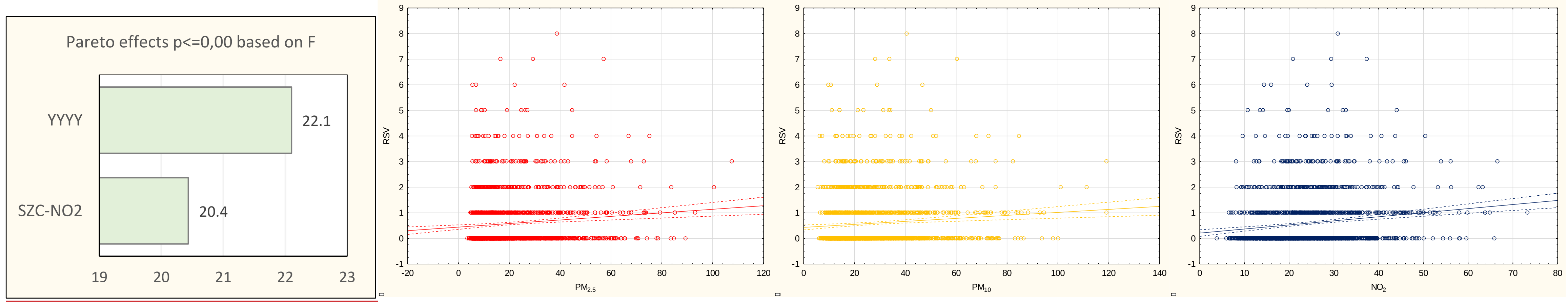

G) Walbrzych

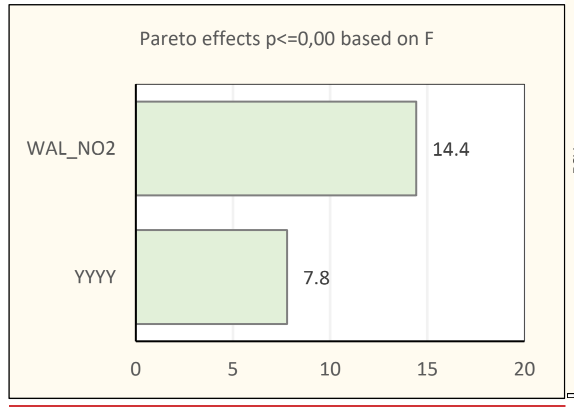

Pareto effects p<=0,00 based on F
WAL_NO2
14.4
YYYY
7.8
0
5
10
15
20

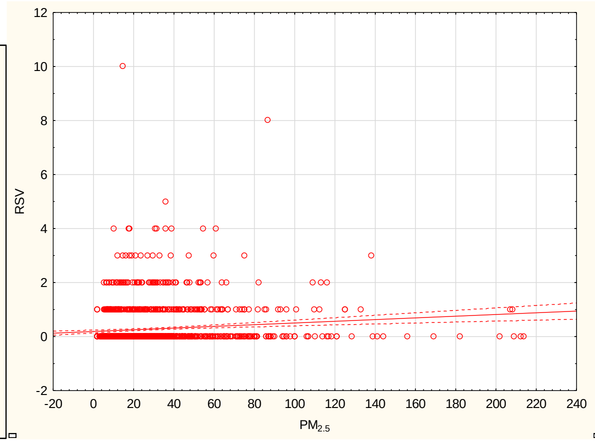

RSV
PM2.5

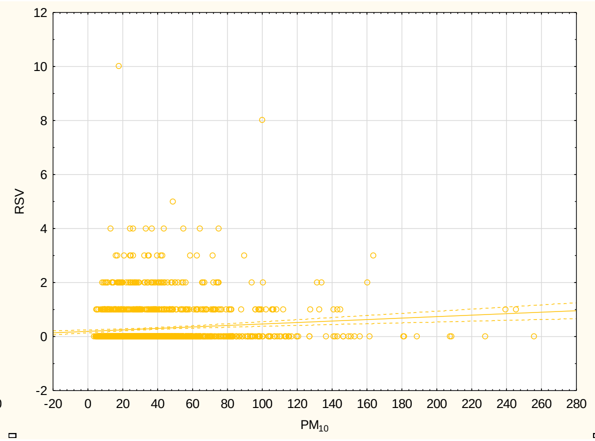

RSV
PM10

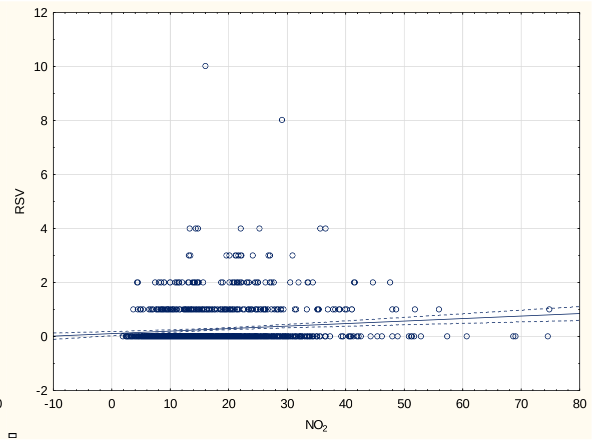

RSV
NO2
